# A highly potent CD73 biparatopic antibody blocks organization of the enzyme active site through dual mechanisms

**DOI:** 10.1074/jbc.RA120.012395

**Published:** 2021-01-13

**Authors:** James E. Stefano, Dana M. Lord, Yanfeng Zhou, Julie Jaworski, Joern Hopke, Tara Travaline, Ningning Zhang, Karen Wong, Amanda Lennon, Timothy He, Eva Bric-Furlong, Cornishia Cherrie, Tristan Magnay, Elisabeth Remy, William Brondyk, Huawei Qiu, Katarina Radošević

**Affiliations:** 1 Biologics Research, Sanofi R&D Framingham, Massachusetts, USA; 2 Translational Sciences, Sanofi R&D, Cambridge, Massachusetts, USA; 3 Sanofi R&D, Vitry, France

**Keywords:** CD73, antibody engineering, enzyme mechanism, enzyme structure, structure-function, protein complex, aggregation, protein crosslinking

## Abstract

The dimeric ectonucleotidase CD73 catalyzes the hydrolysis of AMP at the cell surface to form adenosine, a potent suppressor of the immune response. Blocking CD73 activity in the tumor microenvironment can have a beneficial effect on tumor eradication and is a promising approach for cancer therapy. Biparatopic antibodies binding different regions of CD73 may be a means to antagonize its enzymatic activity. A panel of biparatopic antibodies representing the pairwise combination of 11 parental monoclonal antibodies against CD73 was generated by Fab-arm exchange. Nine variants vastly exceeded the potency of their parental antibodies with ≥90% inhibition of activity and subnanomolar EC_50_ values. Pairing the Fabs of parents with nonoverlapping epitopes was both sufficient and necessary whereas monovalent antibodies were poor inhibitors. Some parental antibodies yielded potent biparatopics with multiple partners, one of which (TB19) producing the most potent. The structure of the TB19 Fab with CD73 reveals that it blocks alignment of the N- and C-terminal CD73 domains necessary for catalysis. A separate structure of CD73 with a Fab (TB38) which complements TB19 in a particularly potent biparatopic shows its binding to a nonoverlapping site on the CD73 N-terminal domain. Structural modeling demonstrates a TB19/TB38 biparatopic antibody would be unable to bind the CD73 dimer in a bivalent manner, implicating crosslinking of separate CD73 dimers in its mechanism of action. This ability of a biparatopic antibody to both crosslink CD73 dimers and fix them in an inactive conformation thus represents a highly effective mechanism for the inhibition of CD73 activity.

CD73 (ecto-5′-nucleotidase, NT5E) is a glycosylated 125-kDa homodimeric membrane-bound enzyme which dephosphorylates AMP in the extracellular milieu to adenosine (1). Adenosine has potent immunosuppressive effects in the tumor microenvironment so CD73 has attracted wide interest as a target for cancer therapy (1, 2, 3, 4, 5, 6). CD73 expression is associated with resistance to anti-HER2 therapy (7), poor prognosis with reduced anti-tumor immune response in a variety of tumor types (1), and the increased growth of tumor cells, migration, and invasion *in vitro* (8). A number of clinical studies are in progress with CD73-specific antibodies (9, 10) and small molecule inhibitors (10, 11), alone or in combination with A2a adenosine receptor antagonists and antibodies to other targets, particularly the PD-1/PD-L1 axis (12). MEDI9447 (oleclumab), a CD73-specific internalizing antibody with moderate inhibition of enzymatic activity, has shown some clinical efficacy as a monotherapy and in combination with the PD-L1 blocker durvalumab (13). There are also indications that CD73 antibodies can exert effects independent of adenosine production. One study indicated that the enhancement of the immune response was mediated through FcγRIV engagement in mice (14) and other work suggested a role for CD73 internalization at suppressing metastasis (10, 13, 15). Nonetheless, adenosine levels in tumors can reach micromolar concentrations, so incomplete inhibition of CD73 activity may be a limiting factor for the efficacy of current CD73-targeting therapeutics (16). Thus, the mechanism by which CD73 affects cancer progression may be complex, suggesting the need for very potent inhibition of enzymatic activity or a combination of mechanisms to achieve optimal efficacy.

The CD73 monomer, with N- and C-terminal domains that are connected through a flexible α-helical linker, is expressed at the cell surface attached to C-terminal glycosylphosphatidylinositol anchor. In the physiological form two monomers associate through extensive noncovalent contacts between the C-terminal domains forming a dimer (17, 18). The active site in each monomer of CD73 is comprised of substrate contact residues in both the N- and C-terminal domains in addition to zinc cofactors bound by the N-terminal domain (18). Following binding of the AMP substrate to the C-terminal domain, the N-terminal domain and zinc cofactors align with the AMP in a “closed” CD73 conformation in which catalysis takes place to generate the adenosine product (19). A large lateral rotation of the N-terminal domain to re-expose the substrate binding site in the “open” conformer then allows product release (18). A limited solvent access to the active site in the closed conformer indicates that cycling between the two forms is required for substrate binding and product release, *i.e.* efficient enzymatic activity (18).

In our hands, obtaining potent inhibition of CD73 enzymatic activity (*e.g.* both a high percentage inhibition and a low EC_50_) with monospecific CD73 antibodies proved challenging, an experience apparently shared by others (20, 21, 22). We decided to examine the possible advantage of a biparatopic antibody approach because of the potentially additive effect of combining antibody specificities. To identify biologically relevant mechanisms of inhibition, we assayed CD73 activity on cells using a highly sensitive LC-MS–based method (23). Our results demonstrate that antibodies can exhibit potent CD73 inhibition when combined in biparatopic variants provided they bind nonoverlapping epitopes on CD73. As a result of this investigation, we discovered one antibody (TB19) that was able to synergize with half of the antibodies to form highly potent biparatopic variants. The structures of CD73 in the complexes with both TB19 and a partner TB38, which combine to form a particularly potent biparatopic, show CD73 in conformations not reported previously and provide further insights into the catalytic mechanism. Our analyses show that the activity of this potent biparatopic variant is provided by a dual mechanism of directly blocking formation of the catalytically active conformer in a complex stabilized by interactions with other CD73.

## Results

### Generation of biparatopic antibodies

We generated a panel of biparatopic antibodies against CD73 using Fab-arm exchange (cFAE) representing the pairwise combinations of 11 parental antibodies unrelated by sequence and previously showing >50% inhibition of CD73 activity in cell-based assays. Each Fab was expressed as a fusion with human IgG1 Fc containing either the F405L or K409R mutation, which destabilize the parental Fc and stabilize the Fc of the biparatopic duobody product (24, 25, 26). Parental antibodies were expressed in small-scale cultures, purified using protein A, and recombined by Fab arm exchange (26). Production of the desired products was verified by capillary isoelectric focusing (cIEF) (Fig. S1). Out of 121 (11 × 11) possible combinations, 88 biparatopic variants were generated that covered all possible combinations in at least one orientation. Eleven monospecific parental antibodies were also reconstructed as comparators by combining the parental F405L and K409R Fc variants to control for possible effect of the Fc mutations on antibody structure and function. In addition, 21 pairings were generated in both Fc orientations to control for possible positional effects of the mutations.

### Inhibition of cellular CD73 by parental and biparatopic antibodies

**Figure 1:**
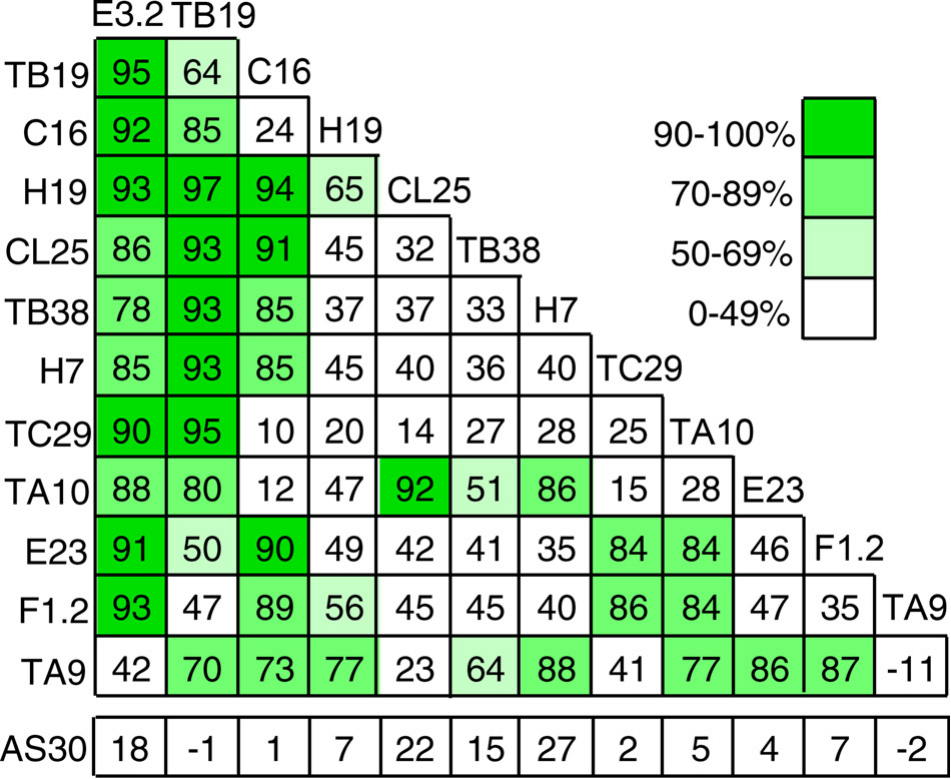
**Screen of inhibitory activity against CD73 on COR-L23 cells.** The inhibition of CD73 activity (%) was determined following exposure to antibodies for 4 h using a LC-MS–based assay with a heavy-isotope AMP substrate (*white shading*: 0–49% inhibition at 1 ug/ml; *pale green*: 50–69% inhibition; *green*: 70–89% inhibition; and *dark green*: 90–100% inhibition). Each *square*, except the furthest right in each row, represents a biparatopic produced by the combination of parental antibodies indicated on the horizontal and vertical axes. The *furthest right square in each row* represents the parental bivalent anti-CD73 antibody reconstructed using Fab-arm exchange. The bottom row (*AS30*) indicates pairings with an irrelevant antibody AS30, to produce monovalent versions of the parental antibodies. Values represent the mean inhibition at 1 μg/ml observed with four replicates of single analyte dilutions.

Purified parental and biparatopic antibodies were tested for potency at 1 μg/ml on COR-L23 lung carcinoma cells expressing human CD73, and the product adenosine quantitated by a LC-MS–based assay (23). The percentage of inhibition of CD73 enzymatic activity by the biparatopics at 1 ug/ml is shown in Fig. 1. Although the extent of inhibition varied widely, most of the biparatopic combinations exhibited higher potency than either parental antibody in the form of a duobody. A number of the parental antibodies yielded highly potent daughter biparatopic variants showing ≥90% inhibition when combined with more than one other antibody. Of these, TB19 and E3.2 formed the highest number of variants with ≥90% inhibition and several of the TB19 pairs, including those with E3.2, H19, TB38, or TC29, achieved ≥95% inhibition. The TB19 and E3.2 antibodies also combined with several other antibodies to achieve ≥80% inhibition. Although both these antibodies showed this promiscuous pairing capability, they were distinguished from each other by complementarity in their pairing patterns. No major differences in the extent of inhibition were observed between biparatopic variants tested in both Fc orientations (in total 16), indicating that the position of the duobody mutations in the Fc did not significantly influence the outcome. To assess whether both parental Fabs were necessary for potency, the parental antibodies were also crossed with an irrelevant antibody (AS30) to create monovalent variant IgGs with only a single Fab capable of interacting with CD73. All of these antibodies showed negligible potency, demonstrating that the Fabs from two cognate parentals must participate (Fig. 1).

### Assessment of bivalent binding

We compared the binding of several of the monovalent antibodies to the biparatopic variants of which they were a part to assess whether the higher potency of the biparatopic was because of additional interactions with CD73. Antibodies were bound to the support and binding to soluble CD73 dimer in solution determined using SPR. Antibody loading was reduced to the lowest feasible level to minimize individual CD73 dimers interacting with more than a single antibody on the support (see under “Experimental procedures”). However, 9 of the 11 biparatopics displayed biphasic dissociation kinetics (Fig. S8), although largely as a consequence of a minor fraction (≤15%) of a faster-dissociating component. In one case (H19/C16) the abundance of this component was larger and similar to that of the monovalent parent C16/AS30 (31% *versus* 38%), suggesting heterogeneity of the C16 monoclonal used for producing both. TA9/AS30 showed a similar heterogeneity (29% lower stability) that was not reflected in the biparatopic daughter TA9/H7. The kd values and their abundances are presented in Table S2 and the half-times for dissociation compared with the monovalent parentals are shown in Fig. S10. In 8 of 11 cases, the kd of the principal dissociation component was within 2.2-fold of the monovalent parent having the highest stability. In contrast, the kds for the monovalent parentals differed by an average of 15-fold (range 1.5- to 73-fold, median 6.2) suggesting in these cases CD73 is bound by a single parental Fab arm on the immobilized antibody. However, in three cases (E3.2/TB19, CL25/TB19, and H19/TB19) the interaction with the biparatopic was significantly more stable than with either monovalent parental (5.4-, 8.8-, and 26-fold, respectively) suggesting the presence of additional contacts with the biparatopic.

Biphasic kinetics of association were also apparent from the limited period provided for binding manifested by a rapid increase in RU immediately following injection followed by a significant decline in rate after 100s. Projection of the RU expected at early times from the rate after 100s assuming pseudo first-order kinetics showed a residual consistent with a fast component binding with first-order kinetics which contributed a significant fraction to the RU (30–49%). A reiterative process to fit both components yielded a combined fit within ± 0.2 of the observed RU over 90% of the course of binding (Fig. S8). Similar to dissociation, the ka value for each of the two components was within 3-fold of a monovalent parent (2.04 ± 1.4-fold, range 1.02–2.71) in contrast to an average ∼6-fold difference between them (5.9 ± 2.1, Table S2), suggesting they reflect the independent binding of CD73 by each parental Fab arm. The ka using a Langmuir 1:1 model was within 30% of the average of the two components (Table S2). Because each component ka could not be unequivocally assigned either one for dissociation, the affinities of the biparatopic and monovalent parents for CD73 were compared using an average *K_D_* value combining the kd of the principal dissociation component with the ka based on Langmuir 1:1 binding. As for dissociation, the apparent affinity of the biparatopic variants (*K_D_*) was similar to those of the more affine monovalent parental antibodies, suggesting the interaction of the biparatopics could be largely attributed to binding of a single Fab arm. In two cases also seen by comparing complex stabilities (CL25/TB19 and TB19/H19) the biparatopic variant showed a significant increase over that of either monovalent parental (26- and 69-fold, respectively). This increase was specific to those combinations because the parents (TB19, H19, CL25) did not produce a similar enhancement with other partners. Because these increases required two cognate arms, we infer that this reflects the interaction of both arms of these two biparatopic variants with CD73, similar to the conclusion arrived at from the dissociation kinetics. However, in the majority of cases the affinity for CD73 was not increased by the addition of a second cognate Fab arm despite its being necessary for potency, suggesting interaction of the biparatopic antibody with an additional CD73 is required for potent inhibition on cells.

### Potency of biparatopics and parental mixtures against cellular CD73

**Figure 2:**
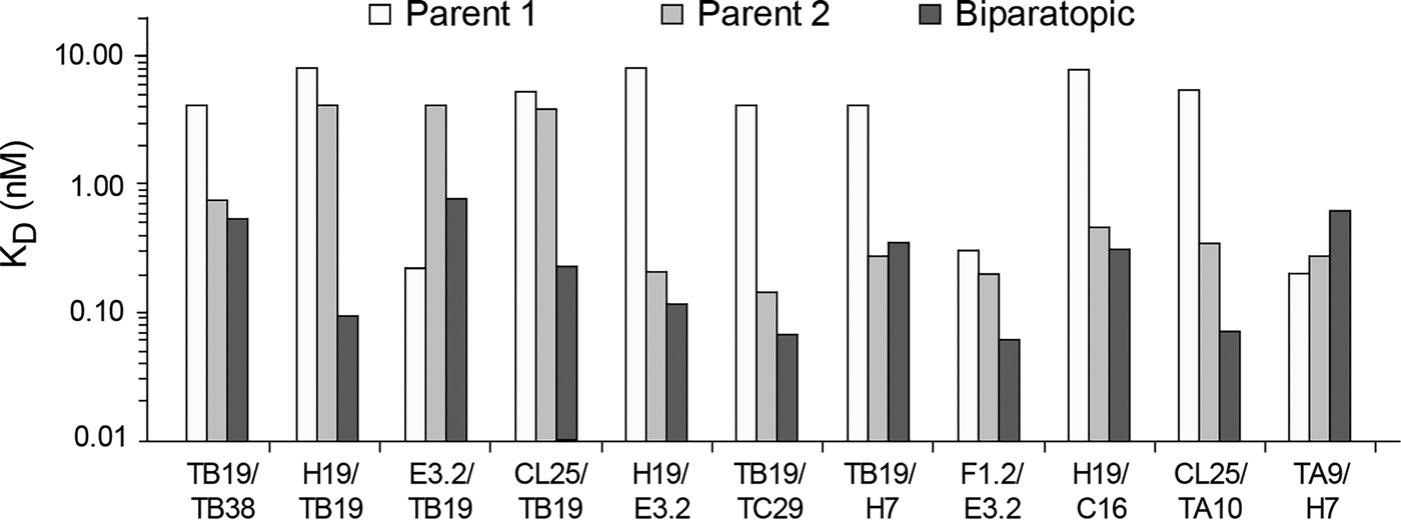
**Relative affinities of parental and biparatopic antibodies for CD73.** Each parental antibody in monovalent form having a second irrelevant arm (AS30) and the biparatopic variant were immobilized and exposed to soluble CD73 in the flow. *K_D_* values are based on association rate constants using a 1:1 Langmuir binding model combined with dissociation rate constants based on either a 1:1 Langmuir model or the principal more stable, component of dissociation where biphasic dissociation kinetics were observed. Raw sensorgrams can be found in Fig. S8, S9.

To further evaluate the benefit of combining the parental antibodies in biparatopic format we determined the EC_50_ and maximum inhibition at saturating antibody concentrations for the most active biparatopics along with their parental mAbs, either alone or in a mixture on COR-L23 cells (Table 1 and Fig. S2). In agreement with the results in Fig. 1, each biparatopic was more potent than either of their two parental antibodies, which showed only partial inhibition up to 10 nm. EC_50_ values for all the biparatopics were in the range of 0.2–0.8 nm. In most cases, the mixtures of parental antibodies yielded similar maximal inhibition as the biparatopics, but in half of the tested combinations, the biparatopic variant in addition showed a lower EC_50_. In the most striking case (TB19/TC29), the biparatopic showed an EC_50_ at least 40-fold lower than the antibody mixture despite the similar apparent affinity for the TC29 monovalent parent and the biparatopic for CD73 (Fig. 2). Strikingly, the very high affinities obtained for a number of the biparatopics based on kds derived from the principal dissociation component seen by SPR were not replicated in low EC_50_s, suggesting some interactions with CD73 in solution may not be fully accessible with CD73 on the cell surface, possibly because of proximity to the membrane or differences in conformational states. In only a single case (CL25/TA10) was the mixture more potent (∼4-fold), indicating that interactions with CD73 provided by that mixture could not be replicated with the biparatopic antibody.

**Table 1:** Potency of biparatopic antibodies and parental mixtures against CD73 on COR-L23 cells. EC_50_ and maximum extents of inhibition are based on nonlinear regression analysis (see “Experimental procedures”)

	Biparatopic		Parental Mix	
Parentals	EC_50_ (nm)	Max. inhibition^a^	EC_50_ (nm)	Max. inhibition^a^
TB19/TB38	0.777	100%	0.841	106%^b^
H19/TB19	0.382	98%	0.629	98%
E3.2/TB19	0.443	97%	0.811	98%
CL25/TB19	0.619	97%	0.636	109%^b^
H19/E3.2	0.224	96%	0.283	99%
TB19/TC29	0.264	95%	13.0	137% b
H7/TB19	0.270	95%	0.541	95%
F1.2/E3.2	0.305	93%	0.256	97%
H19/C16	0.239	93%	0.863	77%
CL25/TA10	0.266	91%	0.073	95%
TA9/H7	0.229	66%	0.658	80%


a maximum inhibition.

b extrapolated value.

### Epitope binning

**Figure 3:**
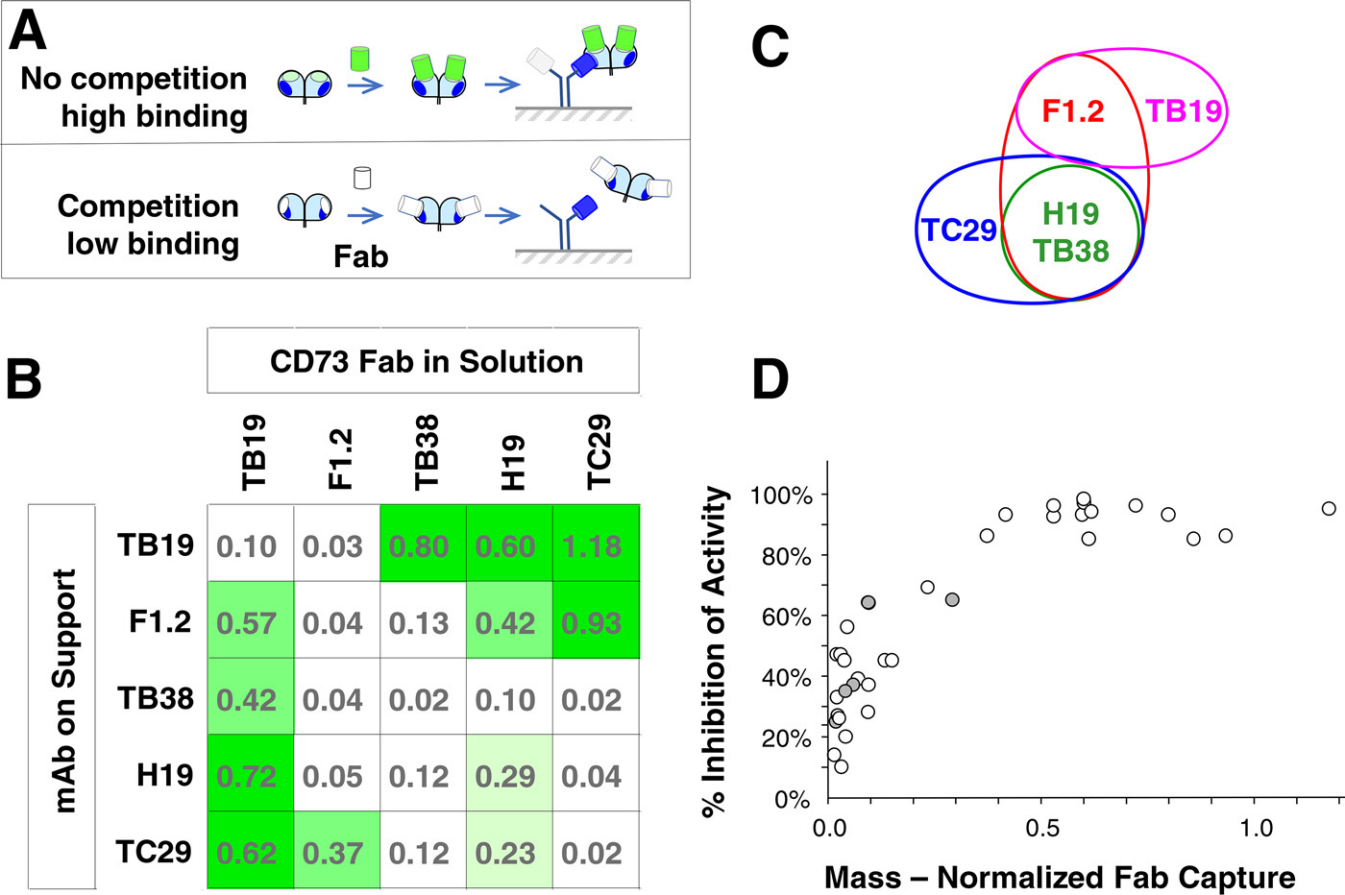
**Epitope binning by biolayer interferometry (Octet).** A mixture of CD73 with a molar excess of Fab was incubated with monovalent parental antibodies immobilized on a solid support. *A*, cartoon of the assay format showing the condition of nonoverlapping epitopes and no blocking (*top panel*) or overlapping epitopes producing complete blocking of capture (*bottom panel*). *B*, capture of CD73/Fab complexes by immobilized antibodies. Capture was normalized to the signal from CD73 alone in the absence of Fab as described under “Experimental Procedures.” Raw Octet traces can be found in Fig. S9. *C*, epitope binning based on the inhibition of capture. *D*, inhibition of CD73 activity on COR-L23 cells *versus* the ability of antibodies to capture a CD73/Fab complex *in vitro*. *Gray-filled circles*: capture of a CD73/Fab complex of the same antibody on the support (parental pair).

Epitopes of the parental antibodies with the highest number of highly potent combinations (TB19, E3.2, TB38, H19, and E3.2) were binned using biolayer interferometry (Fig. 3*A*). In this approach, monovalent IgG antibodies were used to coat the solid support for capturing CD73 mixed with competitor Fabs.

The result of interrogating a subset of the parental antibodies is shown in Fig. 3*B* and Fig. S9. Higher values indicate capture of CD73 bound by the challenge Fab with no/low competition for binding (*i.e.* that the Fab binds to a CD73 epitope not overlapping with that of the coated antibody) whereas lower values reflect blocking of the epitope by the Fab for capture by the immobilized antibody. Allocation of the antibodies to different epitope bins based on these results is shown in Fig. 3*C*. One of the bins contained TB38, H19, and the mostly overlapping TC29, all of which showed susceptibility to each of the Fabs except TB19. However, these three also showed differences in their susceptibilities to competition by different Fabs. For example, the capture of CD73 by a monovalent TB38 IgG was more susceptible to competition by H19 Fab than the capture either by TC29 or H19, whereas TC29 was distinguished from the other two by its partial resistance to competition by the F1.2 Fab, which was unique among all of the antibodies. Although the bins were in most cases clearly delineated, intermediate levels of inhibition were also observed in several cases (H19+H19, TC29+H19, TC29+F1.2, TB19+H19, F1.2+H19, and F1.2+TB19), possibly reflecting partially overlapping epitopes (27) and/or significant differences in affinity. E3.2 could not be binned because of its aspecific interaction with the solid support.

Capture of a CD73::Fab complex by antibody in this binning experiment, reflecting a lack of competition between the parental antibodies, showed a high correlation with inhibition of cellular CD73 enzymatic activity by the corresponding biparatopics (Fig. 3*D*). Pairings of antibodies where more than 35% capture of a Fab was detected invariably produced ≥85% inhibition at 1 μg/ml as a biparatopic and, conversely, combinations with less than 35% capture achieved less than 70% inhibition as a biparatopic. These data indicate that to achieve high potency, the antibodies comprising the biparatopic need to bind nonoverlapping epitopes on CD73.

### Structures of the TB19 and TB38 Fabs in complex with CD73

We expected that analysis of complexes of the IgGs with CD73 might reveal the basis conferring the higher degree of inhibition seen with the biparatopics. However, we were unsuccessful in our attempts to obtain homogeneous complexes of any of the key biparatopic IgGs with CD73 that might be suitable for structure analysis because of formation of heterogeneous mixtures of very high MW products. Several parental antibodies described above (*i.e.* TB19, E3.2) had the interesting property of being able to pair promiscuously to yield potent biparatopics (Fig. 1). Of those, TB19 was of particular interest as its epitope appeared unique and many of its daughter biparatopics showed near-complete inhibition of CD73 activity (Figure 1, Figure 2, Figure 3*B*), suggesting it contributes to potency through a unique and highly synergistic mechanism. Although two antibodies when paired with TB19 showed a high affinity for soluble CD73 consistent with a bivalent interaction (H19, CL25), those were not reflected in a comparably low EC_50_ on cells, suggesting a bivalent interaction might not be easily achievable with the membrane-bound protein. TB38, when combined with TB19 achieved the highest level of inhibition on cells, possessed an unambiguously nonoverlapping epitope which partially or completely overlapped that of other parentals yielding strong inhibition when combined with TB19. Thus, we chose to examine by structure analysis the interactions of the TB19 and TB38 Fabs, as exemplifying a broader class of potent biparatopics which might be useful against both soluble and cellular CD73.

We were able to obtain separate structures of human CD73 complexes with both the TB19 as well as the TB38 Fabs. Production of crystals suitable for diffraction analysis was facilitated by use of the extracellular domain of human CD73 (residues 27–549) deglycosylated with PNGase F. The PNGase F–treated CD73 showed a molecular weight (MW) of 118 kDa by SEC-MALS, which is slightly larger than the polypeptide MW (116 kDa). This was attributable to a glycan observed in the solved structures at position Asn-311, which was thus not susceptible to PNGase F cleavage. Crystallographic parameters of data collection and structure refinement are shown in Table S1.

**Figure 4:**
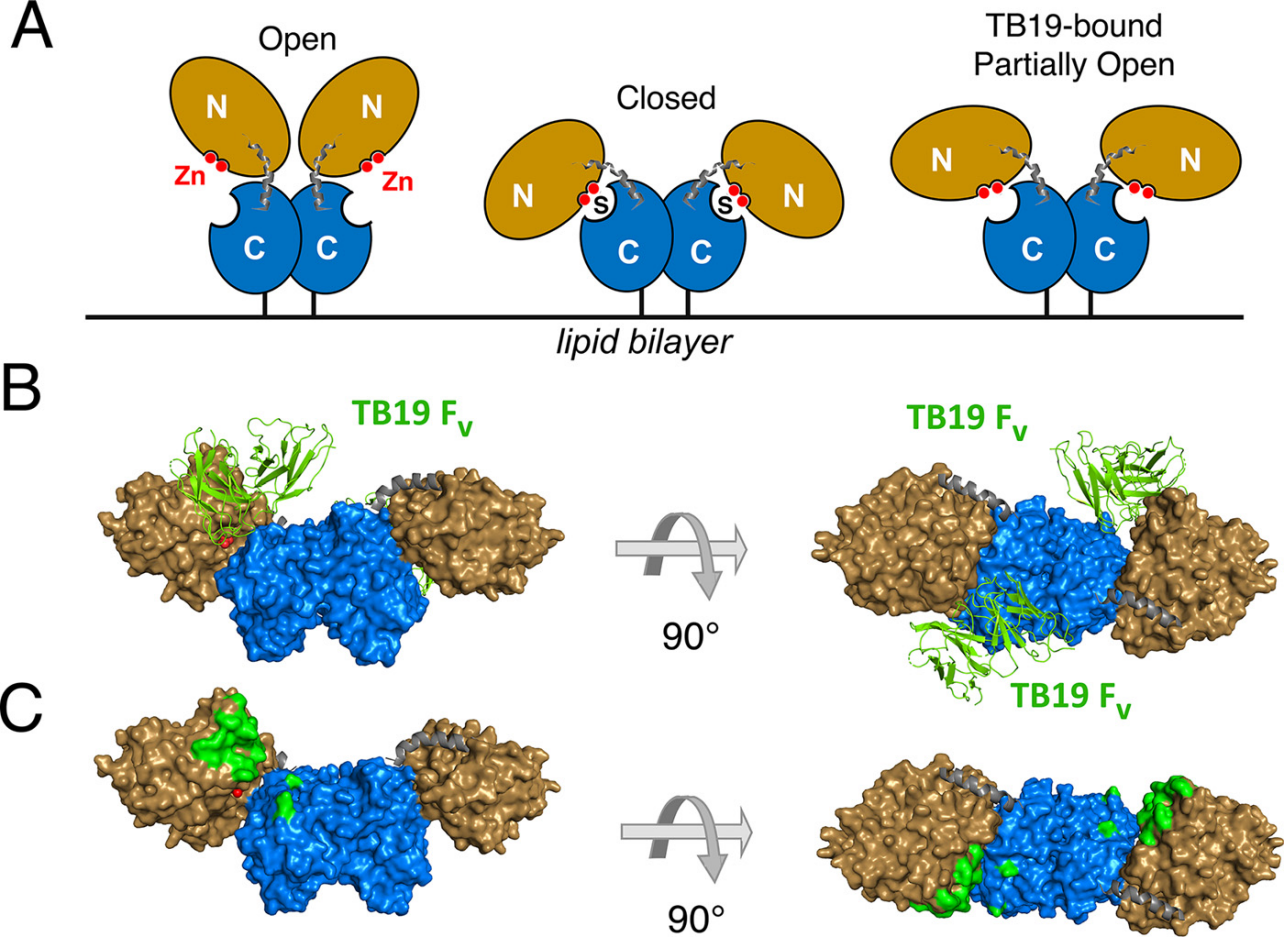
**Structure of TB19 with CD73.***A*, cartoon representation of the different conformational states of CD73. The CD73 N-terminal domain of CD73 is shown in *beige* and the C-terminal domain is shown in *blue*. *Gray coils*: linker elements connecting the two domains. *Red spheres*: zinc cofactor(s) bound by the N-terminal domain. *S*: substrate. Note that the structure of the complex with TB19 was obtained in the absence of substrate, which is not shown. *B*, two TB19 Fv domains binding one CD73 dimer in the intermediate conformation viewed from two different angles. Colors are as in A with the zinc and phosphate molecules shown as *red spheres*. The TB19 Fab (*green*) is shown in a cartoon representation. *C*, mapping of the epitope for TB19 on CD73. The coloring scheme is as *B*; residues interacting withTB19 are shown in *green*.

The structure of CD73 in complex with the TB19 Fab is shown in Fig. 4 and Fig. S3. In the crystal asymmetric unit, one TB19 is bound to one CD73 monomer and only the Fv of the Fab could be built because of weak electron densities in the CH1/CL domains. A biological assembly of dimeric CD73 complex was obtained through a 2-fold crystallographic symmetry operation. In the resulting structure, CD73 is dimerized through an interface between the C-terminal domains (Fig. 4*B*), which closely resembles that of published structures (17, 18).

Within CD73 in the complex with TB19, well-defined positive densities are observed in the active site in the N-terminal domain. Two zinc ions and one phosphate were built accordingly and coordinated by residues Asp-36, His-38, Asp-85, Asn-117, His-118, His-220 and His-243 in the catalytic center (Fig. S7) as the TB19 complex was crystallized in the presence of phosphate. These zinc ions and phosphate are in the same position as the two zinc ions and the β-phosphonate of the substrate analog AMPCP in the closed conformer structure of CD73 (PDB ID 4H2I) (Fig. S3). The conserved dimerization interface and position of the zincs and phosphate indicate the structure of the CD73 dimer in the complex with TB19 is biologically relevant.

CD73 has been previously reported in either an open or a closed conformation, depending on the absence or presence of substrate in the active site, respectively (18) (Fig. 4*A*). However, when bound by TB19, CD73 takes on a conformation in which the N- and C-terminal domains are in an intermediate position between those previously reported for the open and closed conformers (Fig. 4 and Fig. S6A). When the C-terminal domains of earlier structures and TB19-bound CD73 are superimposed, the position of the zinc-coordinating residue His-220 in the N-terminal domain is ∼22 Å away from its position in the closed conformer (PDB ID 4H2I) and 27 Å away from that in the open conformer (PDB ID 4H2F; see also Fig. S6C).

All of the TB19 CDR loops except CDRL2 contact a portion of the N-terminal domain adjacent to the zinc and phosphate binding site (Fig. S3), although none of the antibody residues directly interact with any of the catalytic center forming residues. In addition, the TB19 CDRH2 residue Ser-62 and CDRL1 residue Ser-26 (Fig. 4, *B* and *C*) are spatially close to the C-terminal domain, but 20 Å away from the substrate binding residues including Arg-354, Asn-390, Arg-395, Phe-417, Phe-500, and Asp-506. In the presence of TB19 those substrate-binding residues are far from the catalytic center and the zincs in the N-terminal domain. For example, the residues Phe-417 and Phe-500 which bind the adenine ring are 11–13Å from their positions in the closed conformer with substrate (PDB ID 4H2I).

Because of the orientation of TB19 and its epitope location, clashes between C-terminal domain and TB19 are observed when superimposing the N-terminal domains of CD73 in our structure and the closed conformer of CD73 (Fig. S3). Thus, bound TB19 will block the alignment of N- and C-terminal domains in CD73 and prevent formation of the closed conformer. As a result, TB19 binding will separate the zinc ions and catalytic residues of the N-terminal domain from the phosphoanhydride bond of the substrate, thereby blocking enzymatic activity.

**Figure 5:**
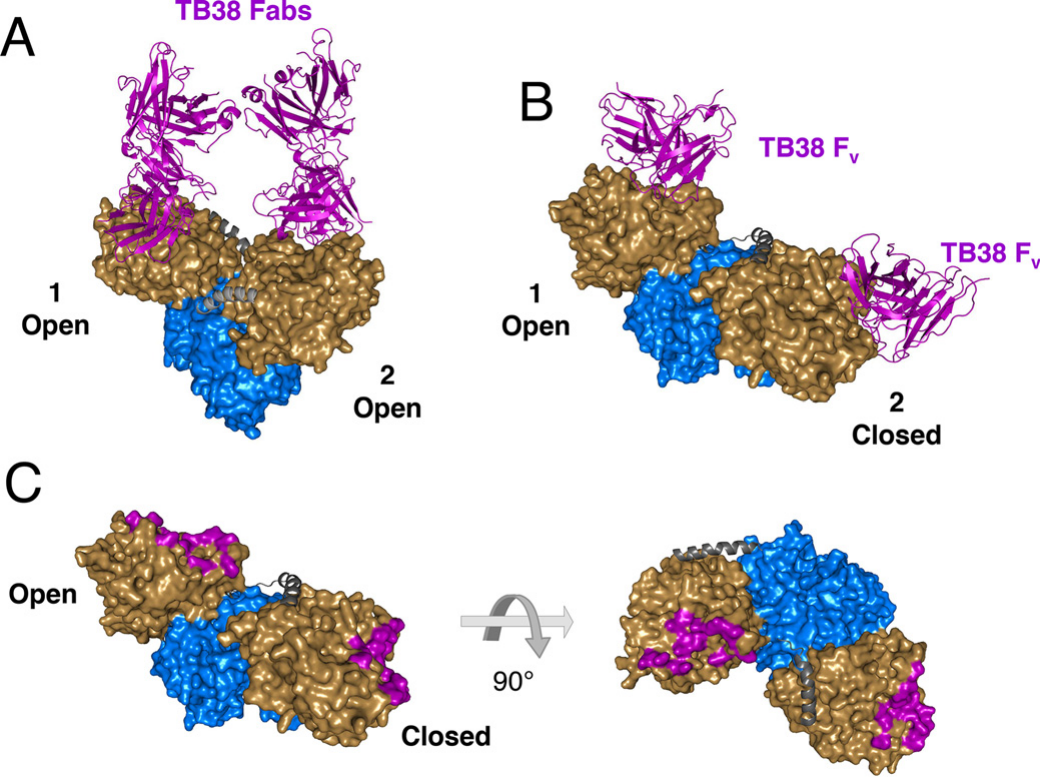
**Structures of TB38 with CD73.** CD73 coloring scheme: *beige* = N-terminal domain, *blue* = C-terminal domain, *gray* = linker. *A*, TB38 Fab::CD73 structure with CD73 in the open conformation (chains A and B in PDB ID 6VCA). Numbers identify each monomer TB38 Fab is shown in a *purple* cartoon representation. *B*, TB38 Fv::CD73 structure with the CD73 in an open/closed hybrid conformation (chains C and D in PDB 6VCA). Monomer 1 is in the open conformation and monomer 2 in the closed conformation (see text). TB38 Fv is shown in *purple*. *C*, mapping of the TB38 epitope residues (*purple*) on CD73 in the open/closed hybrid conformation as shown in (*B*).

In contrast to TB19, the TB38 Fab and CD73 yielded structures with each asymmetric unit containing two CD73 dimers in different conformations with all of the monomers bound by one Fab (Fig. 5). In the first structure (Fig. 5*A*), electron densities for the CH1/CL domains were well-defined and the full Fab structure could be built. In the second (Fig. 5*B*), weak density for the constant domains was observed so only the Fv domains were built. Strikingly, the conformation of CD73 in the two structures is quite different. In the first, CD73 is in a symmetrical open conformation which can be superimposed on the canonical open conformer in PDB ID 4H2F with a root mean square deviation value of 1 Å. However, the CD73 dimer in the second structure is in a nonsymmetrical conformation not reported previously in which the monomers are in different conformations (Fig. 5*C*). In this hybrid structure, one monomer is in the open conformation previously observed in a crystal with bound adenosine (PDB ID 4H2F) while the other is in the closed conformation seen in the presence of the substrate analog AMPCP (PDB ID 4H2I) (18). In both complexes, the TB38 Fab contacts residues solely in the N-terminal domain (including Lys-145, Ser-152, Ser-155, Gly-156, Leu-159, Lys-162, Glu-203, Lys-206, Leu-210, and Asn-211) and all six CDRs are engaged in the interactions. Mapping of the epitope residues of TB19 and TB38 on the partially open structure of CD73 (Fig. S4) and by sequence alignment (Fig. S5) show that the epitopes are non-overlapping, albeit in close proximity in agreement with the binning results.

**Figure 6:**
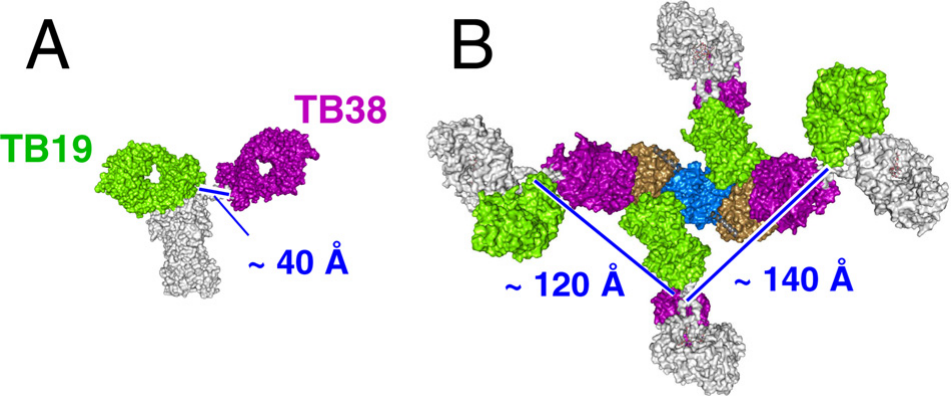
**Potential modes of co-engagement of CD73 by the TB19/TB38 biparatopic.** Bispecific antibodies are modeled based on TB19:CD73, TB38:CD73, and full-IgG1 (PDB 1ZHZ) structures. *A*, surface representation of a TB19/TB38 biparatopic antibody. *Arrow*: distance between the last residues in the CH1 domains. The TB19 Fab is shown in *green*, the TB38 Fab in *purple*. The Fc is shown *light gray*. *B*, a model of four TB19/TB38 biparatopic antibodies bound by a CD73 dimer in the partially open configuration, as seen for the complex with TB19. The N-terminal domains of CD73 are shown in *beige* and the C-terminal domains in *blue*. *Arrow*: distances separating the last residues of the CH1 domains of the opposing Fabs in adjacent biparatopic antibodies.

To assess possible engagement of CD73 dimer by a bispecific TB19/TB38 antibody, the IgG was modeled by replacing the Fvs of a complete IgG antibody structure (PDB ID 1HZH) with those of TB19 and TB38 (Fig. 6). The distance between the CH1 domains of TB19 and TB38 in this model (Fig. 6*A*) is ∼40 Å (measured between the Cα of Ala-225 of the CH1 domain). We were unable to model bivalent binding to CD73 in the partly open conformation by this biparatopic IgG binding either of the two epitope pairs on the same or opposing monomers, although each CD73 monomer could be bound by two antibodies monovalently as illustrated in Fig. 6*B*. In order for a single antibody to bind the CD73 dimer bivalently, the C-terminal residue of the Fab CH1 domains would need to be separated by ∼120Å and ∼140Å to bind the epitopes either on the same or opposite monomers respectively, which is much further than can be achieved by an IgG. We conclude it is likely that a biparatopic TB19/TB38 antibody would be incapable of binding a single CD73 dimer in a bivalent manner.

## Discussion

Strategies for creating biparatopics often rely on rational design to partner well-characterized parental antibodies with the intent of obtaining hybrid molecules combining the desirable properties of each parent. Small camelid antibodies (28) and non-antibody scaffolds such as affibodies (29) have most frequently been employed because of the larger number of potential bivalent interactions and the relative ease for designing the bispecific engagement (30). Although more limited, even monoclonal antibodies and larger formats such as tetravalent IgGs have displayed similarly enhanced potency as a consequence of target crosslinking and internalization (13, 20, 31), which could also apply to a biparatopic IgG. A biparatopic CD73 IgG combining a weakly inhibiting but internalizing mAb with a potent but noninternalizing antibody was shown to combine the beneficial properties of both (22).

In the present study, we paired CD73-specific antibodies preselected on the basis of detectable CD73-inhibitory activity in cell-based assays and their sequence dissimilarity but without any prior understanding of their mechanism of action and/or epitope specificity. Although a similar combinatorial approach can be executed recombinantly, the present approach starting with purified parental antibodies using Fab arm exchange provided the facile generation of a large number of molecules. We were then able to combine this with a high-throughput assay to screen for biparatopic combinations to obtain the most potent inhibition of cellular CD73 activity. In the course of this analysis we identified a number of potent biparatopic variants which shared a single parental antibody, TB19, and were capable of fully inhibiting CD73 activity at low nanomolar concentrations. As a monoclonal, TB19 itself exhibited only modest affinity and could only achieve a high degree of CD73 inhibition at a submicromolar concentration which typically would make it unattractive as a candidate even for a biparatopic pairing. However, its capacity to successfully partner with many other antibodies illustrates that combining such weakly potent monoclonal antibodies can provide unexpected synergies arising from complementary mechanisms of action which acting alone are only modestly effective. Thus, in general, a combinatorial approach applying less-restrictive criteria for selecting parental antibodies may be the most fruitful for identifying useful biparatopics. However, we have also found that pairing antibodies which do not compete for the same epitope provided the highest degree of inhibition even for this dimer target, so epitope information can be useful for limiting the number of parentals to be recombined in the case of large antibody sets.

The structure of the CD73::TB19 complex demonstrates that bound TB19 Fab blocks the alignment of the N- and C-terminal domains with substrate which is necessary for catalysis. The “locking” of CD73 by antibody binding to this intermediate partly open inactive conformer presumably reflects the primary mechanism of action of this antibody. The high EC_50_ may reflect an inability to access to its epitope on CD73 in some conformational states, as shown by the clash with the C-terminal domain if bound to its epitope residues on the N-terminal domain of CD73 in a closed conformation (Fig. S3). In addition, the conformation of CD73 in the TB19 structure is consistent with an intermediate state between the open and closed conformations, which reflect a rotation of the N-terminal domain by 140° (Fig. S6), a process which occurs at a rate approaching 50 s^−1^ (32). Thus, it is not unexpected for a high antibody concentration to be required to drive binding to such an intermediate to block activity.

In contrast to TB19, TB38 is bound by CD73 in the open, closed, and even a surprising hybrid conformation in which one monomer is in the open and the other in the closed conformation. In the recently reported crystal structure of a complex of CD73 and Fab IPH5301, both CD73 monomers are in the closed conformation (33). However, the hybrid conformer of CD73 when bound by TB38 has not been reported previously. Although we cannot rule out an influence of crystal packing in producing this structure, it suggests an unappreciated conformational flexibility to the CD73 dimer such that the energy barrier for one subunit to transition between conformations while the other is held in a fixed position is not insurmountable. In that case, the two monomers of the CD73 dimer might be capable of independently undergoing the conformational changes required for catalysis rather than acting in concert to maintain structural symmetry. The presence of TB38 bound to this structure also raises the possibility that TB38 even when bound might permit CD73 conformational changes required for catalysis which would be consistent with the low level of inhibition observed for the monovalent TB38 antibody (15%) despite its nanomolar affinity (Fig. 2 and Table S2). The increased inhibition by the parental TB38 antibody with the added arm capable of interacting with CD73 compared with its monovalent version may reflect an ability to crosslink CD73 dimers, which is consistent with our inability to model bivalent binding of the TB38 IgG on the CD73 dimer based on the structures of the Fab complexes. The requirement that the TB38 epitopes on neighboring CD73 dimers be available in a position for crosslinking might make such inhibition particularly sensitive to competition by excess free antibody in solution, to yield flat or reduced potency at higher concentrations (“hook” effect) as observed with several of the monoclonals (Fig. S2) and as reported previously (20). However, a freedom for TB38 to bind CD73 in a range of conformations, as suggested by the structures, might at the same time improve the chances for the antibody to be suitably oriented for crosslinking. Such a lack of preference of TB38 for a specific CD73 conformation may be the source of the particularly high degree of inhibition of its biparatopic with TB19 which distinguishes TB38 from other parentals.

How the combination of the TB19 and TB38 Fabs, that on their own act through different but only modestly effective mechanisms, leads to the highly potent activity of the biparatopic is not completely clear. Based on our modeling of the biparatopic IgG structure, we know it is highly unlikely that both Fab arms would be able to bind the TB19/TB38 epitope pair on either the same or opposing monomers of the same CD73 dimer, implicating interdimer crosslinking in the mechanism of action, consistent with the weak inhibition achieved by monovalent antibodies that are incapable of crosslinking, The synergism of TB19 and TB38 is most easily explained by binding of CD73 on the cell membrane being driven by the more affine TB38 arm which then provides a TB19 Fab to capture and lock neighboring CD73 dimers in the intermediate conformation. Although the freedom of movement of the N-terminal domain of the TB38-bound CD73 may not be impaired by its binding, its motion would still be constrained by tethering to an adjacent CD73 which has been locked in intermediate conformation by interaction with the TB19 Fab. Thus, although the role of TB38 may largely be to facilitate crosslinking, TB19, in addition to collaborating with TB38 in crosslinking neighboring CD73, will also block formation of the catalytically active conformer and thus act in two ways to inhibit CD73 activity.

Whereas monoclonal antibodies can form chains with dimer targets, biparatopics additionally have the capability to form crosslinked networks by branching, enabled by the binding of both epitopes on a monomer by separate antibodies as shown in the structure model of TB19/TB38 (Fig. 6*B*). Crosslinked networks will be stabilized on account of those additional interactions to more effectively promote otherwise weak interactions such as that with TB19. Positioning of CD73 dimers brought together in a nascent crosslinked network can also promote antibody binding to make network formation a cooperative process, as is suggested by the steep dose-responses for many of the biparatopics (Fig. S2). The proximity of epitopes in a network that are available for crosslinking would also act to minimize monovalent binding and thus suppress “hook” effects, whereas even loosely crosslinked networks could entrap CD73 dimers to accelerate binding. Although we have not solved the structures of CD73 in complex with other parental antibodies which synergize with TB19, we speculate that these alternative partners could play a similar role as TB38 in forming networks to promote the TB19 interaction and thus facilitate its inhibitory activity. The high frequency of successful pairings with TB19 suggests that this role can be fulfilled by a variety of antibodies capable of inhibiting CD73 as monoclonals through crosslinking, as long as they do not compete for the TB19 epitope.

In conclusion, our investigations demonstrate that the combinatorial screening of biparatopic combinations has the ability to identify antibodies with synergistic mechanisms of action and provides a path to identify highly potent combinations of interest as therapeutics. A similar approach may serve to illuminate the structure-function relationships of antibodies with many other therapeutic targets.

## Experimental procedures

### Materials

Mercaptoethylamine, adenosine 5′-(α,β-methylene) diphosphate (AMPCP), adenosine, formic acid, Triton X-100, dithiobis-2,4-dinitrophenol, and Pharmalyte 3-10 were obtained from Sigma-Aldrich. RPMI 1640 medium was from Thermo Fisher Scientific. [^15^N]_5_AMP was obtained from Silantes GmbH (no. 123303801). Isoelectric focusing reagents (methyl cellulose, arginine, pI markers, anolyte, and catholyte) were obtained from ProteinSimple.

### Generation of (biparatopic) antibodies

CD73-specific monoclonal antibodies were isolated using common mouse immunization and phage display approaches using soluble human CD73 as antigen (data not shown). Twelve sequence-unrelated parental antibodies with IC_50_ in the range of 1–25 nm and with at least 50% inhibition of CD73 in cell-based assays at saturating concentrations of antibody were selected for the study. Bispecific variants were produced using a modification of a published Duobody procedure (24) except using microdialysis for product purification. Equimolar amounts of F405L and K409R Fc variants of each parental huIgG1 (25–50 μg each) were combined in a total volume of 90 μl PBS to which 10 μl 7.5 M mercaptoethylamine pH 7.4 was added. The mixture was incubated 4h at 30°C in a forced-air incubator, transferred to individual cassettes taken from 96-well dialysis plate strips (Pierce) and subjected to three rounds of dialysis (1 h, 1.5 h, and overnight) at room temperature. For more than six samples, the reactions were transferred to dialysis cassette strips mounted on a carrier plate. The plate was suspended over a reservoir and transferred between reservoirs containing fresh PBS after each round of dialysis. After the second dialysis, total free thiol in the retentate was below the limit of detection using dithiobis-2,4-dinitrophenol. The final products were stored at 4°C. Product formation was determined by cIEF. Parental antibodies for analysis were reconstructed by crossing the F405L and K409R parents in the same fashion as the test duobodies.

### Characterization of biparatopic antibodies

Formation of the duobody products of the cFAE reaction was determined using a cIEF (Maurice, Protein Simple, San Jose, CA, USA). This approach was chosen because the pI of the bispecific daughter molecules would be expected to fall between that of each of the two parents. To increase the relative contribution of charge differences in the CDRs and frameworks, cIEF was performed on Fab′_2_ fragments obtained by IdeZ digestion of the cFAE products. The cFAE product (4 μl 1 mg/ml) was mixed with 4 μl 1 unit/μl IdeZ (Fabricator Z, Genovis) in water and mixed by trituration. The tubes were incubated 4 h at 37°C in an air incubator followed by addition of 36 μl 1.1× Pharmalyte methylcellulose/ampholine mixture, mixed and centrifuged 4 min at 13 kG. The supernatant (30 μl) was transferred to a 96-well plate for analysis. Samples were loaded on a cIEF cassette for 55 s and focused for 1.5 min at 1.5 kV then 6 min at 3 kV. Resolved products were detected by fluorescence. Formation of the desired duobody product was assessed by the disappearance of the parental antibody Fab′2 peaks and formation of a Fab′2 peak with a pI near the average of the two parental Fab′2 along with the absence of a F405L parental Fc peak at ∼pI 7.6. The duobody Fc fragment with both mutations (F405L:K409R) was not resolved from the K409R parent, likely because of a limited change in the p*K_a_* of the arginine in the environment surrounding this residue. The IdeZ focused at pI 7.14 and below. An example result is shown in Fig. S1.

### Analysis of biparatopic binding

The ability of the biparatopics to engage CD73 bivalently (*e.g.* at two epitopes) was determined by comparing the affinity of the biparatopic to each parental antibody in monovalent form by use of surface plasmon resonance (SPR). Antibody at low density was on the support and CD73 in the flow. SPR was performed on a Biacore T200 instrument (GE Healthcare) at 25°C using HBS-EP+ (10 mm HEPES, 150 mm NaCl, 3 mm EDTA, 0.05% (v/v) surfactant P20, pH 7.4) as running buffer and Protein A series S sensor chips (GE Healthcare). Antibodies diluted to the lowest concentration providing reliable determination of kinetic constants (between 5 and 30 RU) were loaded using a 30 s injection at 10 μl/min. Following washing, CD73 (at 3, 12, or 32 nm) was then passed over the surface for 5 min at 30 μl/min. HBS-EP+ was then applied and dissociation followed for 30 min. The sensor surface was regenerated with 10 mm glycine-HCl pH 1.5 for 30 s at 20 μl/min. Kinetic constants were calculated using a Biacore T200 Evaluation software (GE Healthcare). A 1:1 Langmuir binding model was used except for cases in which bivalent fits using BiaEvaluation software showed lower apparent residuals raising the possibility of biphasic binding. In those cases, kd values of each component during dissociation were determined beginning by fitting the longer *t*_1/2_ component to a first-order decay defined by the kinetics after 1000 s. An exponential fit to the residual for that component between 100 and 200 s was used to calculate the abundance and kd for rapidly dissociating component(s). The criterion that the interval used for fitting the slower component begin after a minimum of four times the *t*_1/2_ of the rapidly dissociating component was applied. The components of association were separately derived by initially fitting the approach to saturation (RUmax) within a window from 100 s out to a point a minimum of 0.2 RU from the RUmax as a first-order reaction for a range of assumed RUmax values. The best fit parameters and RUmax were then used as a starting point for further refinement. The positive residual between observed RU and this fit extended to earlier times was treated as an independent pseudo first order reaction reflecting a rapid-binding component. A reiterative process varying the rate constants and fraction of each component with the level of binding (RU) after 300 s was used to obtain fits within 0.2 of the observed RU and a near-zero slope for the net residual over the 300-s measurement. Variation testing showed the values were true R^2^ minima for the overall fit. The RUmax had a negligible effect on the rate constants or fraction of each component. Fits were performed in Excel.

### CD73 inhibition (potency) cell-based assay

Potency of the biparatopics was determined using a modification of a previously disclosed method (23). COR-L23 cells expressing CD73 (4 × 10^3^/well) were grown overnight to ∼50% confluence in 40 μl 1640 medium with l-glutamine and 10% heat-inactivated FBS in a 384-well transparent-bottom plate (Greiner Bio One). Antibodies diluted in 1640 medium (10 μl) were added and the plates incubated for 3 h at 37°C. Antibody dilutions and additions were performed on an Agilent Bravo liquid handler. AMPCP (100 μm) was substituted for antibodies as a zero-activity control (23, 34). Substrate (5 μl 200 μm^15^N_5_-AMP, Silantes GmbH, Munich, Germany) was added using a GNF dispenser II (GNF Systems, San Diego, CA, USA) and the plates incubated at 37°C for 1 h. The reactions were then quenched with 5 μl 12% formic acid in 1640 medium and a portion of the quenched reactions (40 μl) was filtered by centrifugation for 30 min at 3,500 × G through a 10-kDa MWCO ultrafiltration plate (Pall Life Sciences). The filtrates were stored at −80°C. The adenosine product was determined by LC-MS/MS analysis as described previously (23). Data were analyzed by nonlinear least squares fits (GraphPad Prism). Activity relative to no-antibody controls in the same plate sector and normalized to the least-squares fit maximum activity (% CNTL) is shown. The results of potency determinations (Table 1) are expressed as the projected maximal % inhibition at saturating antibody concentrations. In initial screening, three concentrations (0.25, 0.5, and 1 μg/ml) were tested in quadruplicate dilution series and the average % inhibition shown (Fig. 1) is based on residual activity at a single concentration (1 μg/ml).

### Epitope binning

Epitope binning of a subset of antibodies was performed using a pre-mix format and biolayer interferometry using a modification of a previously described method (35). In this format, binding of antigen pre-mixed with a molar excess of Fab is compared with the binding of antigen alone. Analysis was performed in 16-channel mode on an Octet QK384 (Pall Life Sciences). Antibodies were bound by protein A biosensors for 5 min, a baseline established for 1 min, then transferred to 100 nm CD73 or 100 nm CD73 with a 4-fold molar excess Fab for 3 min followed by transfer to buffer to follow dissociation for 3 min. All samples were diluted in PBS pH 7.4 containing 0.1% (w/v) BSA and 0.01% (v/v) Tween 20 and the assays were carried out at 30°C. Data were analyzed using the ForteBio Data Analysis 7.1 software (Pall Life Sciences) by taking report points at the end of the association phase. Normalized capture values were calculated by the signal (nm) divided by the signal from CD73 alone times the relative mass of CD73 compared with the CD73::(Fab)_2_ complex (0.56).

### Structure determinations

Recombinant TB19 and TB38 Fab were expressed in Expi293F cells, purified by a CaptureSelect CH1-XL Affinity Matrix (Thermo Fisher), and buffer exchanged into PBS. Human CD73 27-549 was cloned with a C-terminal His_6_-tag and expressed in ExpiHEK293 cells. CD73 was purified using a nickel column, buffer exchanged into PBS, deglycosylated overnight with PNGase F, and further purified using size-exclusion chromatography. The molar mass of the product was determined by SEC on a Superdex 200 column in 150 mm NaCl, 20 mm HEPES, pH 7.0, using multi-angle light scattering (WYATT miniDAWN® Treos and a Wyatt Optilab® T-rEX inline refractometer). Data were evaluated using Wyatt ASTRA 6.1 software. Each respective Fab was then incubated with CD73 on ice for 1 h and loaded on to a Superdex 200 10/300 GL column (GE Healthcare) pre-equilibrated with 20 mm HEPES, pH 7.0, 150 mm NaCl. Fractions corresponding to the eluted complex peak were pooled and concentrated to 9 mg/ml for crystallization trials. TB19 Fab::CD73 crystallized in 0.1 M sodium potassium phosphate pH 6.2, 35% 5-methyl-2,4-pentanediol, and 2.5% pentaerythritol ethoxylate at 4°C. These crystals were cryo-protected in 20% ethylene glycol and mother liquor. X-ray diffraction data were collected at EMBL Hamburg P14 using an Eiger 16M detector. Data were indexed/integrated using XDS and scaled using Aimless (36, 37). Molecular replacement was performed using Phaser (38) and three search ensembles: separated CD73 N- and C-terminal domains (PDB ID 4H2I) and a TB19.3 Fv model generated by MOE (39). TB38 Fab::CD73 produced crystals at 4°C in 1.6 M sodium phosphate monobasic monohydrate, 0.4 M potassium phosphate dibasic, and 0.1 M sodium phosphate citrate pH 5.3. Crystals were flash frozen in liquid nitrogen using 20% glycerol in mother liquor as cryoprotectant. X-ray diffraction data were collected at the European Synchrotron Radiation Facility Beamline ID-30b with a Pilatus 3 6M detector. Data were indexed/integrated using XDS and scaled using Aimless (36, 37). Molecular replacement was performed iteratively using Phaser (38). For the first round of molecular replacement, CD73 monomer (PDB ID 4H2F) and a TB38 Fab MOE-generated model was used as search models for MOE (39). For the second round, the previously found CD73 monomer was separated into its N and C-terminal domains and searched along with the Fv domain alone of TB38. For both structures, model rebuilding was performed in Coot (40) and refinement was completed using Phenix (41) and REFMAC5 (42). Data collection and refinement statistics are listed (Table S1). Software used in this project was accessed through the SBGrid consortium (43). All protein structure images except for Fig. S6C were generated using Pymol (The PyMOL Molecular Graphics System, Version 1.5, Schrödinger, LLC). The alignment of structures in Fig. S6C was performed using BIOVIA Discovery Studio Visualizer (Discovery Studio Modeling Environment, Dassault Systèmes BIOVIA, v.4.5.0.15071).

## Data availability

The structures presented in this paper have been deposited in the Protein Data Bank under accession codes 6VC9 for TB19::CD73 and 6VCA for TB38::CD73. All remaining data are presented in the article.
