# Supplementary material for: A highly potent CD73 biparatopic antibody blocks organization of the enzyme active site through dual mechanisms
Source: J Biol Chem. 2021 Jan 13;295(52):18379–89. doi: 10.1074/jbc.RA120.012395 (PMC7939394; doi:10.1074/jbc.RA120.012395)
Supplement: Supplementary file 1 [file mmc1.pdf]

Supporting Information for

# A highly potent CD73 biparatopic antibody blocks organization of the enzyme active site through dual mechanisms

**James E. Stefano[1§*], Dana M. Lord[1#], Yanfeng Zhou[1], Julie Jaworski[1], Joern Hopke[1], Tara Travaline[◊], Ningning Zhang[1], Karen Wong[1], Amanda Lennon[1], Timothy He[2], Eva Bric-Furlong[1], Cornishia Cherrie[1], Tristan Magnay[1], Elisabeth Remy[1], William Brondyk[1‡], Huawei Qiu[1†] and Katarina Radošević[1§]**

From [1]Biologics Research, [2]Translational Sciences, Sanofi R&D, Framingnam MA, USA; [#]Ohana Biosciences, Cambridge MA, USA; [◊]Fog Pharma, Cambridge MA, USA; [‡]Invetx, Boston MA, USA; [†]Xilio Therapeutics, Waltham MA, USA; [§]Lonza, St. Beauzire, France

Running Title: *Potent biparatopic antibodies against CD73*

To whom correspondence should be addressed: James E. Stefano: Sanofi R&D, Framingnam Massachusetts, USA; james.stefano@sanofi.com. Tel: (1)-508-270-2271.

**Table S1**

**Crystallographic data and refinement statistics**

| | **TB19 Fab CD73 Complex** | **TB38 Fab CD73 Complex** |
|---|---|---|
| **Diffraction data** | | |
| **PDB Code** | 6VC9 | 6VCA |
| **Wavelength (Å)** | 0.9762 | 0.9763 |
| **Unit cell (Å)** | 118.31, 74.22, 148.33 | 236.91, 336.2, 222.15 |
| **Space group** | I 1 2 1 | C 2 2 2 |
| **Resolution range (Å)** [a] | 59.04 - 2.25 (2.33 - 2.25) | 78.61 - 3.73 (3.863 - 3.73) |
| **Data completeness (%)** | 99.6 (99.8) | 99.9 (100) |
| **Redundancy** | 3.8 (3.9) | 6.6 (6.7) |
| **Average** $I/\sigma(I)$ | 10.8 (2.1) | 6.0 (0.6) |
| **CC1/2 (%)** | 99.8 (82.6) | 99.5 (19.4) |
| $R_{merge}$ | 0.071 (0.693) | 0.255 (3.599) |
| **Refinement statistics** | | |
| **Resolution range (Å) a** | 30.00 - 2.25 | 40.00 - 3.73 |
| $R_{work}$ **(%)** | 23.66 | 22.58 |
| $R_{free}$ **(%)** | 27.85 | 26.40 |
| **No. of non-hydrogen atoms** | | |
| **Protein/Glycan** | 5814 / 61 | 27754 / 81 |
| **Zn/PO4/Water** | 2 / 15 / 126 | ---- |
| **B-factors (average)** | | |
| **Protein/Glycan** | 66.13 / 103.37 | 197.39 / 228.12 |
| **Zn/PO4/Water** | 52.34 / 70.96 / 56.09 | ---- |
| **r.m.s.d.** | | |
| **Bond length (Å)** | 0.016 | 0.008 |
| **Bond angle (°)** | 1.757 | 1.682 |
| **Ramachandran plot (%)** | | |
| **Favored** | 93.14 | 89.66 |
| **Allowed** | 5.25 | 7.97 |
| **Outlier** | 1.62 | 2.37 |

[a]Values in parentheses are for the highest-resolution shell.

| | Name | $k_a$ (1/Ms) Langmuir 1:1 | $k_a$ (1/Ms) Biphasic | kd (1/s) Langmuir 1:1 | kd (1/s) Biphasic | $K_D$ (M)* |
|---|---|---|---|---|---|---|
| Parent 1 | **TB19/AS30** | 1.65E+05 | - | 6.90E-04 | - | 4.18E-09 |
| Parent 2 | **TB38/AS30** | 2.37E+05 | - | 1.87E-04 | - | 8.14E-10 |
| Biparatopic | **TB19/TB38** | 1.84E+05 | - | 1.02E-04 | - | 5.55E-10 |
| | | | | | | |
| Parent 1 | **TB19/AS30** | 1.65E+05 | - | 6.90E-04 | - | 4.18E-09 |
| Parent 2 | **H19/AS30** | 6.31E+05 | - | 5.23E-03 | - | 8.29E-09 |
| Biparatopic | **TB19/H19** | 4.49E+05 | 2.81E+05 (65%) | 1.12E-04 | 2.70E-05 (85%) | 6.02E-11 |
| | | | 1.38E+06 (35%) | | 2.70E-03 (15%) | |
| | | | | | | |
| Parent 1 | **E3.2/AS30** | 5.80E+05 | 3.34E+05 (60%) | 1.72E-04 | 7.22E-05 (92%) | 1.25E-10 |
| | | | 1.22E+06 (40%) | - | 1.58E-03 (8%) | |
| Parent 2 | **TB19/AS30** | 1.65E+05 | - | 6.90E-04 | - | 4.18E-09 |
| Biparatopic | **E3.2/TB19** | 3.64E+05 | 1.58E+05 (57%) | 1.64E-04 | 1.28E-04 (91%) | 3.52E-10 |
| | | | 8.50E+05 (43%) | | 4.06E-03 (9%) | |
| | | | | | | |
| Parent 1 | **CL25/AS30** | 5.54E+05 | - | 3.06E-03 | - | 5.52E-09 |
| Parent 2 | **TB19/AS30** | 1.65E+05 | - | 6.90E-04 | - | 4.18E-09 |
| Biparatopic | **CL25/TB19** | 4.93E+05 | 3.24E+05 (70%) | 1.30E-04 | 7.86E-05 (86%) | 1.60E-10 |
| | | | 1.50E+06 (30%) | | 7.06E-03 (14%) | |
| | | | | | | |
| Parent 1 | **H19/AS30** | 6.31E+05 | - | 5.23E-03 | - | 8.29E-09 |
| Parent 2 | **E3.2/AS30** | 5.80E+05 | 3.34E+05 (60%) | 1.72E-04 | 7.22E-05 (92%) | 1.25E-10 |
| | | | 1.22E+06 (40%) | | 1.58E-03 (8%) | |
| Biparatopic | **H19 /E3.2** | 5.74E+05 | 1.06E+06 (61%) | 1.71E-04 | 1.30E-04 (85%) | 2.26E-10 |
| | | | 1.41E+05 (39%) | | 9.98E-03 (15%) | |
| | | | | | | |
| Parent 1 | **TB19/AS30** | 1.65E+05 | - | 6.90E-04 | - | 4.18E-09 |
| Parent 2 | **TC29/AS30** | 7.59E+05 | - | 1.11E-04 | - | 1.47E-10 |
| Biparatopic | **TB19/TC29** | 6.85E+05 | 1.19E+06 (55%) | 8.96E-05 | 7.80E-05 (91%) | 1.14E-10 |
| | | | 1.16E+05 (45%) | | 6.00E-03 (9%) | |
| | | | | | | |
| Parent 1 | **TB19/AS30** | 1.65E+05 | - | 6.90E-04 | - | 4.18E-09 |
| Parent 2 | **H7/AS30** | 6.89E+05 | - | 1.95E-04 | - | 2.83E-10 |
| Biparatopic | **TB19/H7** | 7.52E+05 | 7.03E+05 (55%) | 1.49E-04 | 2.46E-04 (100%) | 3.28E-10 |
| | | | 5.31E+06 (45%) | | | |
| | | | | | | |
| Parent 1 | **F1.2/AS30** | 9.26E+05 | - | 3.12E-04 | - | 3.36E-10 |
| Parent 2 | **E3.2/AS30** | 5.80E+05 | 3.34E+05 (60%) | 1.72E-04 | 7.22E-05 (92%) | 1.25E-10 |
| | | | 1.22E+06 (40%) | | 1.58E-03 (8%) | |
| Biparatopic | **F1.2/E3.2** | 9.39E+05 | 1.60E+06 (60%) | 1.24E-04 | 9.89E-05 (97%) | 1.05E-10 |
| | | | 4.09E+05 (40%) | | 8.59E-04 (3%) | |
| | | | | | | |
| Parent 1 | **H19/AS30** | 6.31E+05 | - | 5.23E-03 | - | 8.29E-09 |
| Parent 2 | **C16/AS30** | 5.74E+05 | 3.54E+05 (57%) | 3.93E-04 | 1.71E-04 (61%) | 2.99E-10 |
| | | | 1.26E+06 (44%) | | 8.66E-03 (38%) | |
| Biparatopic | **H19/C16** | 4.54E+05 | 3.78E+05 (72%) | 3.66E-04 | 1.23E-04 (69%) | 2.70E-10 |
| | | | 1.31E+06 (28%) | | 3.48E-03 (31%) | |
| | | | | | | |
| Parent 1 | **CL25/AS30** | 5.54E+05 | - | 3.06E-03 | - | 5.52E-09 |
| Parent 2 | **TA10/AS30** | 3.40E+05 | - | 1.34E-04 | - | 3.92E-10 |
| Biparatopic | **CL25/TA10** | 5.70E+05 | 8.44E+05 (65%) | 2.20E-04 | 6.14E-05 (92%) | 1.08E-10 |
| | | | 1.88E+05 (35%) | | 5.36E-03 (7%) | |
| | | | | | | |
| Parent 1 | **TA9/AS30** | 5.21E+05 | 1.34E+06 (51%) | 5.13E-04 | 2.70E-04 (73%) | 5.18E-10 |
| | | | 2.65E+05 (49%) | | 1.29E-02 (25%) | |
| Parent 2 | **H7/AS30** | 6.89E+05 | - | 1.95E-04 | - | 2.83E-10 |
| Biparatopic | **TA9/H7** | 5.97E+05 | 2.75E+05 (59%) | 2.19E-04 | 1.80E-04 (91%) | 3.01E-10 |
| | | | 1.60E+06 (41%) | | 1.86E-03 (9%) | |

***Langmuir ka with major component kd**

**Table S2.** Representative SPR kinetic data for assessing bivalent engagement of single CD73. The affinity of the biparatopics for CD73 was compared to that of the parental antibodies in monovalent form. To avoid potential avidity effects from binding of CD73 in solution by separate antibodies on the chip surface, parental antibodies were loaded on the chip at the lowest level sufficient to reliably assess kinetic constants. Data are grouped as shown in Figure 2. Note that kinetic parameters for parental antibodies that are shared between multiple biparatopics are shown in each case to facilitate comparisons. Values represent fits to curves obtained with 3, 12 and 32 nM CD73 in the flow. The

association rate constants in the case of biphasic kinetics are shown with their abundances following a 300s binding phase in parentheses.  Dissociation rate constant abundances are based on the To intercept of the fits to each component.

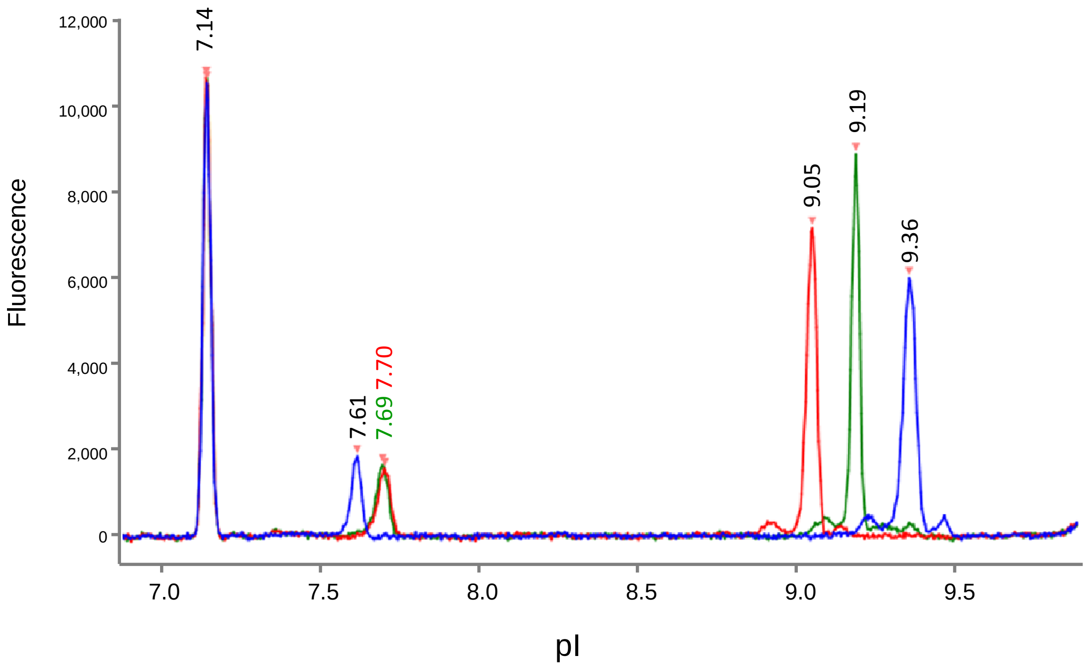


**Figure S1.** Confirmation of biparatopic formation by cIEF. Duobody products and the parental mAbs (4 µg each) were digested with IdeZ to obtain the Fab'2 and Fc and resolved by cIEF. Blue: E3.2(F405L) parental, Red: H19(K409R) parental, Green: cFAE product E3.2/H19. The Fab'2 fragments have pI's above 8.5. The peaks between pI 7.5 and 8 represent the Fc's. The peak at pI 7.1 is IdeZ.

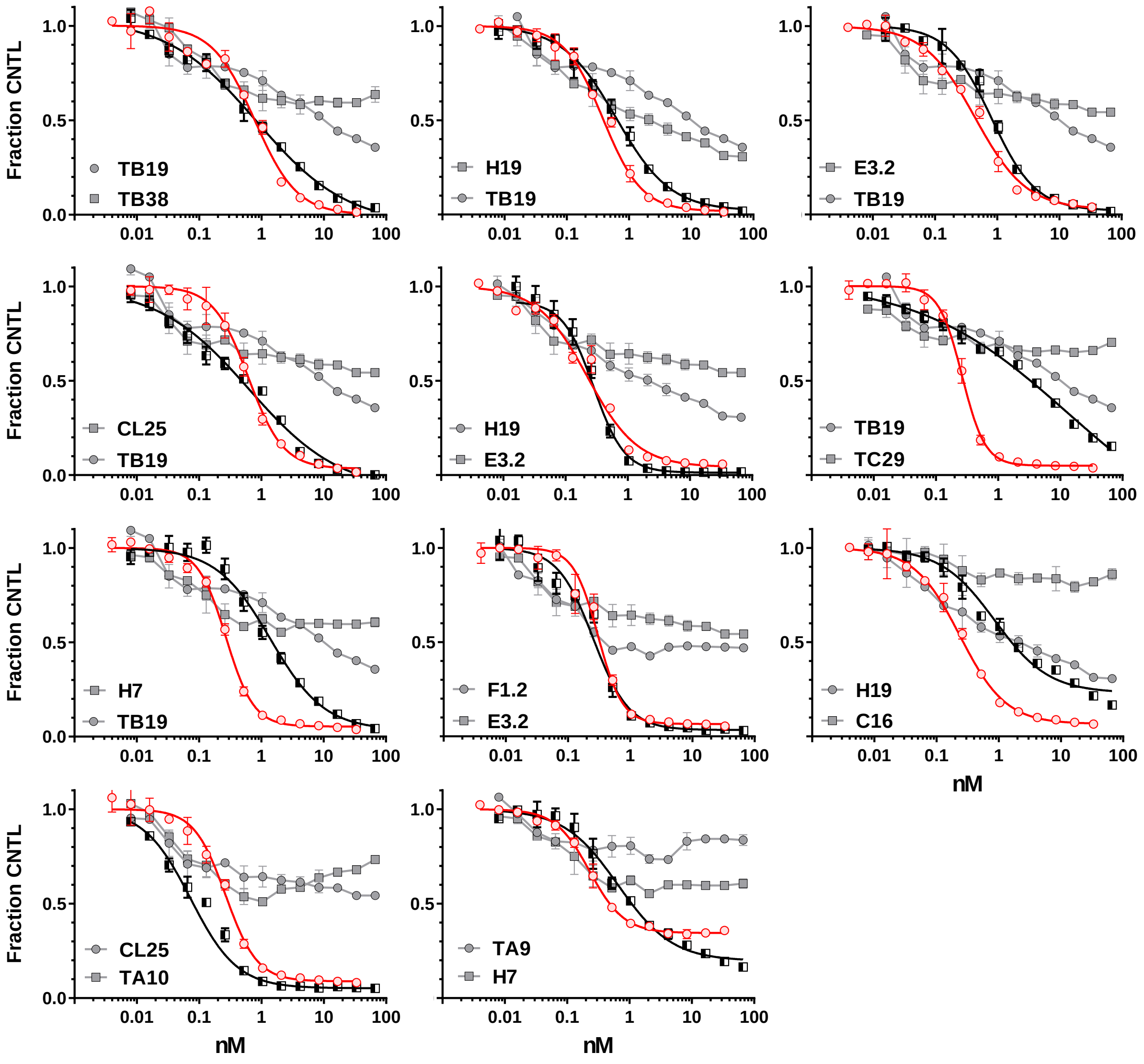


**Figure S2.** Dose-responses for 11 of the most potent biparatopics, parental antibodies and the mixtures of the parentals on COR-L23 cells. The activity (fraction of the no-antibody control) is plotted for the biparatopics (red), parental mixtures (black) and each of two parentals (grey) are shown relative to the total antibody concentration. Error bars reflect the standard deviation of 3 replicate serial dilutions performed robotically from a single sample on a single assay occasion. Data were obtained from assays performed on several occasions. On each occasion, at least one potent duobody from prior determinations was used as a comparator to check reproducibility.

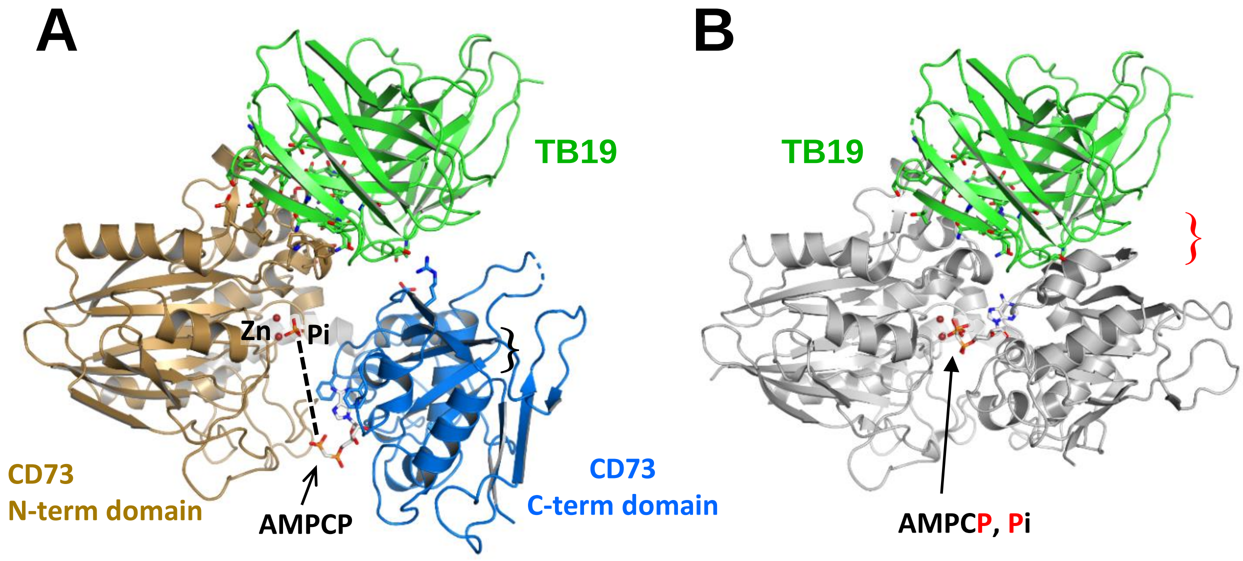


**Figure S3.** Spatial arrangement of the CD73 monomer with the TB19 Fv. **A.** TB19 bound to CD73 in the partially-open conformation. The zinc and inorganic phosphate at the catalytic center are shown as red dots (Zn) and red/orange stick (Pi) respectively. Interacting residues (within 4Å) in the TB19 Fv and the N-terminal domain are shown in stick form. For illustration, the substrate analog AMPCP (in stick form) bound by the C-terminal domain of the closed conformer structure 4H2I is superimposed on the TB19:CD73 structure to show its position and the interacting CD73 residues Phe417 and Phe500. Note that AMPCP is not present in the TB19 structure. **B.** Modeling of TB19 onto the closed conformation structure 4H2I by superimposing the CD73 N-terminal domains of the two structures. The zinc ions and β-phosphonate of AMPCP in 4H2I occupy the same positions as the zinc and inorganic phosphate in the TB19-bound CD73 structure. The TB19 variable region clashes with the C-terminal domain in 4H2I (red bracket).

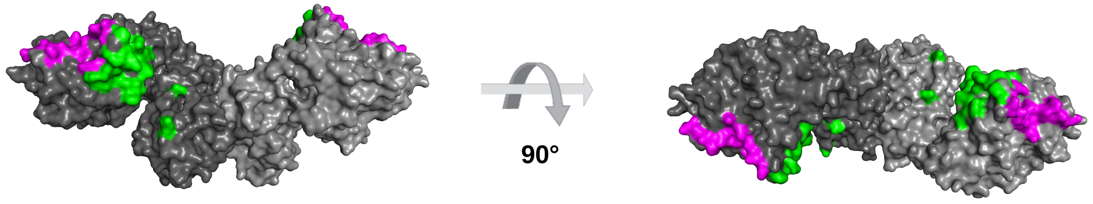


**Figure S4.** The epitopes for TB19 (green) and TB38 (fuschia) mapped onto one subunit of the CD73 homodimer shown in the partially open configuration, as in the TB19 co-crystal structure (dark gray/light gray). TB38 epitope residues common to both the open and closed monomers in the hybrid conformer are shown.

```
               30        40        50        60        70        80
TB19     WELTILHTNDVHSRLEQTSEDSSKCVNASRCMGGVARLFTKVQQIRRAEPNVLLLDAGDQ
TB38     WELTILHTNDVHSRLEQTSEDSSKCVNASRCMGGVARLFTKVQQIRRAEPNVLLLDAGDQ

               90       100       110       120       130       140
TB19     YQGTIWFTVYKGAEVAHFMNALRYDAMALGNHEFDNGVEGLIEPLLKEAKFPILSANIKA
TB38     YQGTIWFTVYKGAEVAHFMNALRYDAMALGNHEFDNGVEGLIEPLLKEAKFPILSANIKA

              150       160       170       180       190       200
TB19     KGPLASQISGLYLPYKVLPVGDEVVGIVGYTSKETPFLSNPGTNLVFEDEITALQPEVDK
TB38     KGPLASQISGLYLPYKVLPVGDEVVGIVGYTSKETPFLSNPGTNLVFEDEITALQPEVDK

              210       220       230       240       250       260
TB19     LKTLNVNKIIALGHSGFEMDKLIAQKVRGVDVVVGGHSNTFLYTGNPPSKEVPAGKYPFI
TB38     LKTLNVNKIIALGHSGFEMDKLIAQKVRGVDVVVGGHSNTFLYTGNPPSKEVPAGKYPFI

              270       280       290       300       310       320
TB19     VTSDDGRKVPVVQAYAFGKYLGYLKIEFDERGNVISSHGNPILLNSSIPEDPSIKADINK
TB38     VTSDDGRKVPVVQAYAFGKYLGYLKIEFDERGNVISSHGNPILLNSSIPEDPSIKADINK

              330       340       350       360       370       380
TB19     WRIKLDNYSTQELGKTIVYLDGSSQSCRFRECNMGNLICDAMINNNLRHTDEMFWNHVSM
TB38     WRIKLDNYSTQELGKTIVYLDGSSQSCRFRECNMGNLICDAMINNNLRHTDEMFWNHVSM

              390       400       410       420       430       440
TB19     CILNGGGIRSPIDERNNGTITWENLAAVLPFGGTFDLVQLKGSTLKKAFEHSVHRYGQST
TB38     CILNGGGIRSPIDERNNGTITWENLAAVLPFGGTFDLVQLKGSTLKKAFEHSVHRYGQST

              450       460       470       480       490       500
TB19     GEFLQVGGIHVVYDLSRKPGDRVVKLDVLCTKCRVPSYDPLKMDEVYKVILPNFLANGGD
TB38     GEFLQVGGIHVVYDLSRKPGDRVVKLDVLCTKCRVPSYDPLKMDEVYKVILPNFLANGGD

              510       520       530       540
TB19     GFQMIKDELLRHDSGDQDINVVSTYISKMKVIYPAVEGRIKFS
TB38     GFQMIKDELLRHDSGDQDINVVSTYISKMKVIYPAVEGRIKFS
```

**Figure S5**. CD73 epitope residues for TB19 and TB38. Residues for TB19 and TB38 are highlighted in cyan and green respectively. The epitope residues for TB38 shown were observed in both the open and closed monomers of the hybrid CD73 conformer bound by TB38.

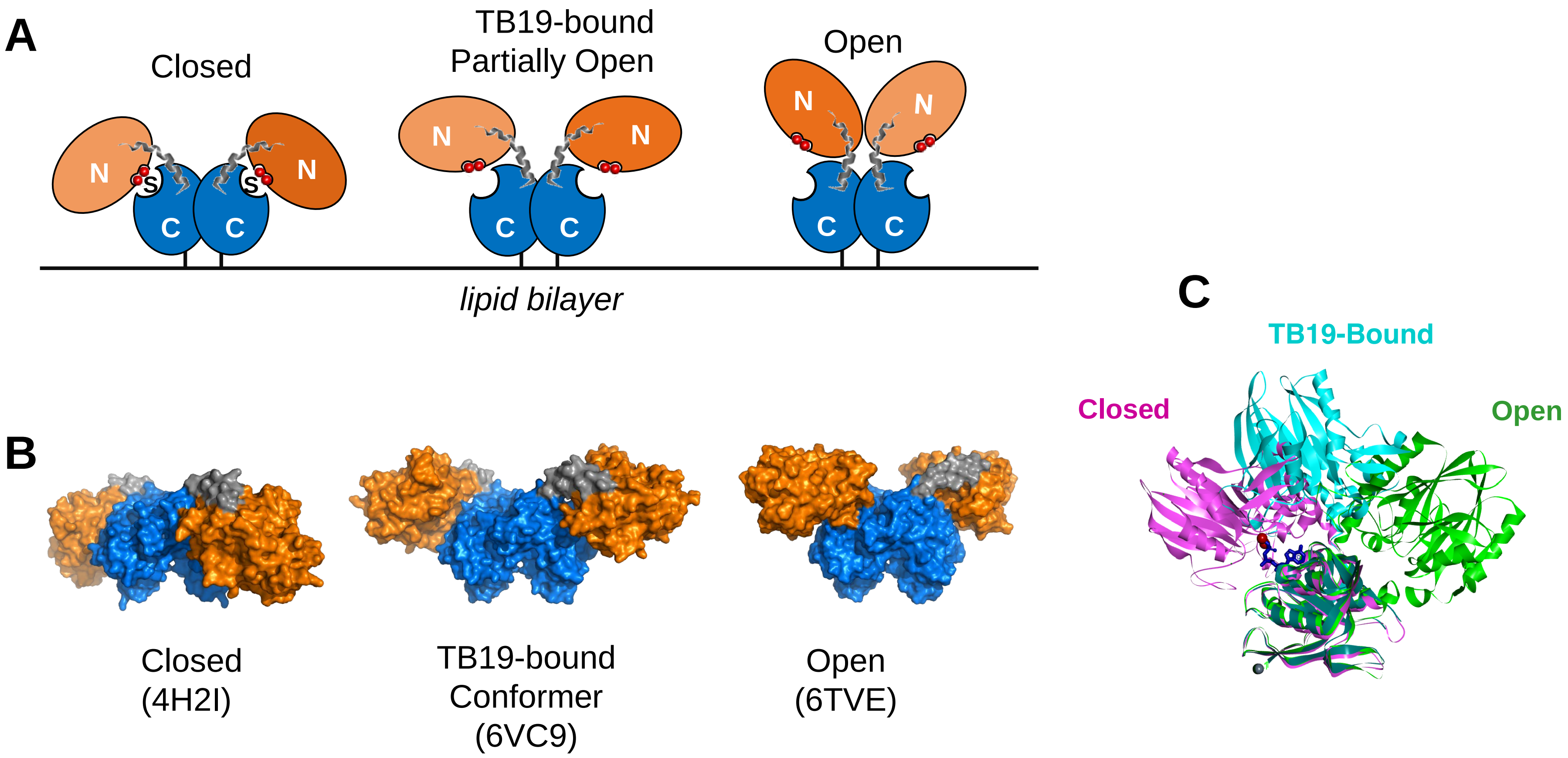


**Figure S6.** CD73 conformers structures associated with the concept cartoons in Figure 4. **A.** Representation of CD73 conformations similar to that shown in Fig4, reflecting the key features of each conformer. Shading is added for comparison to the structures in B. **B.** Actual structural equivalents to the diagrammatic representations in A. Note that the N-terminal domain on the right rotates back into the plane of the page between the open, TB19, and closed configurations. **C.** Structures of a CD73 monomers with the C-terminal domains aligned showing the rotation of the N-terminal domain in each of the 3 conformations: Green (open), Aqua (TB-19) and Pink (closed). The TB19 Fab is not shown for clarity. The view is from below relative to those in B, perpendicular to the plane of rotation of the N-terminal domain. Grey sphere: C-terminal residue of extracellular domain, red spheres: zinc atoms in the closed conformer structure 4H2I, blue sticks: substrate analog AMPCP in 4H2I.

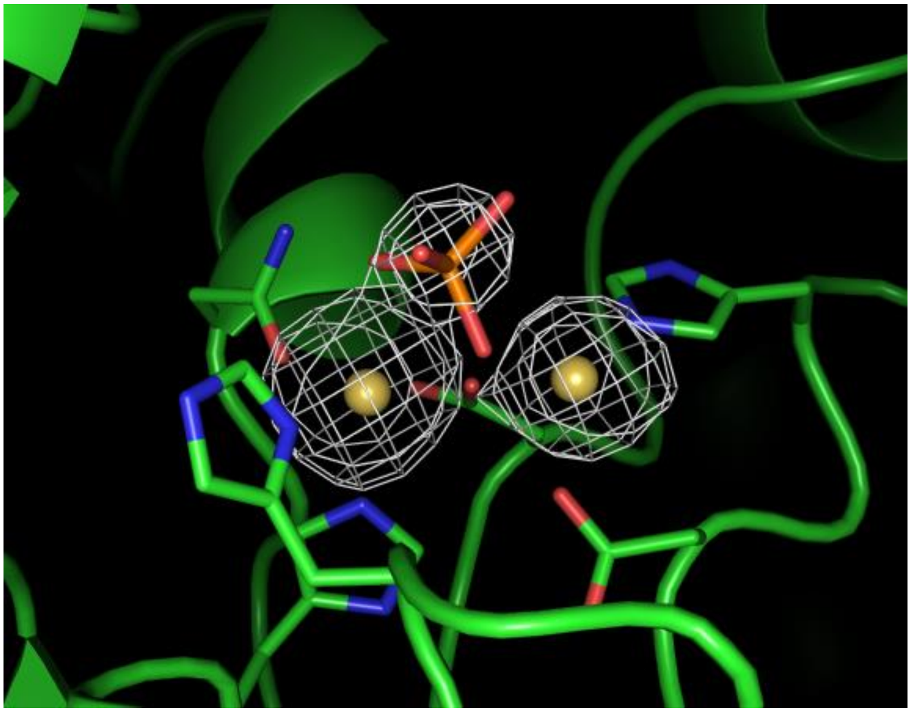

**Figure S7.** Fo-Fc omit map for zincs and phosphate in the N-terminal domain of the structure with TB19 at 5 sigma.  Yellow balls: zincs; red and orange sticks: oxygen and phosphorus respectively of the phosphate ion

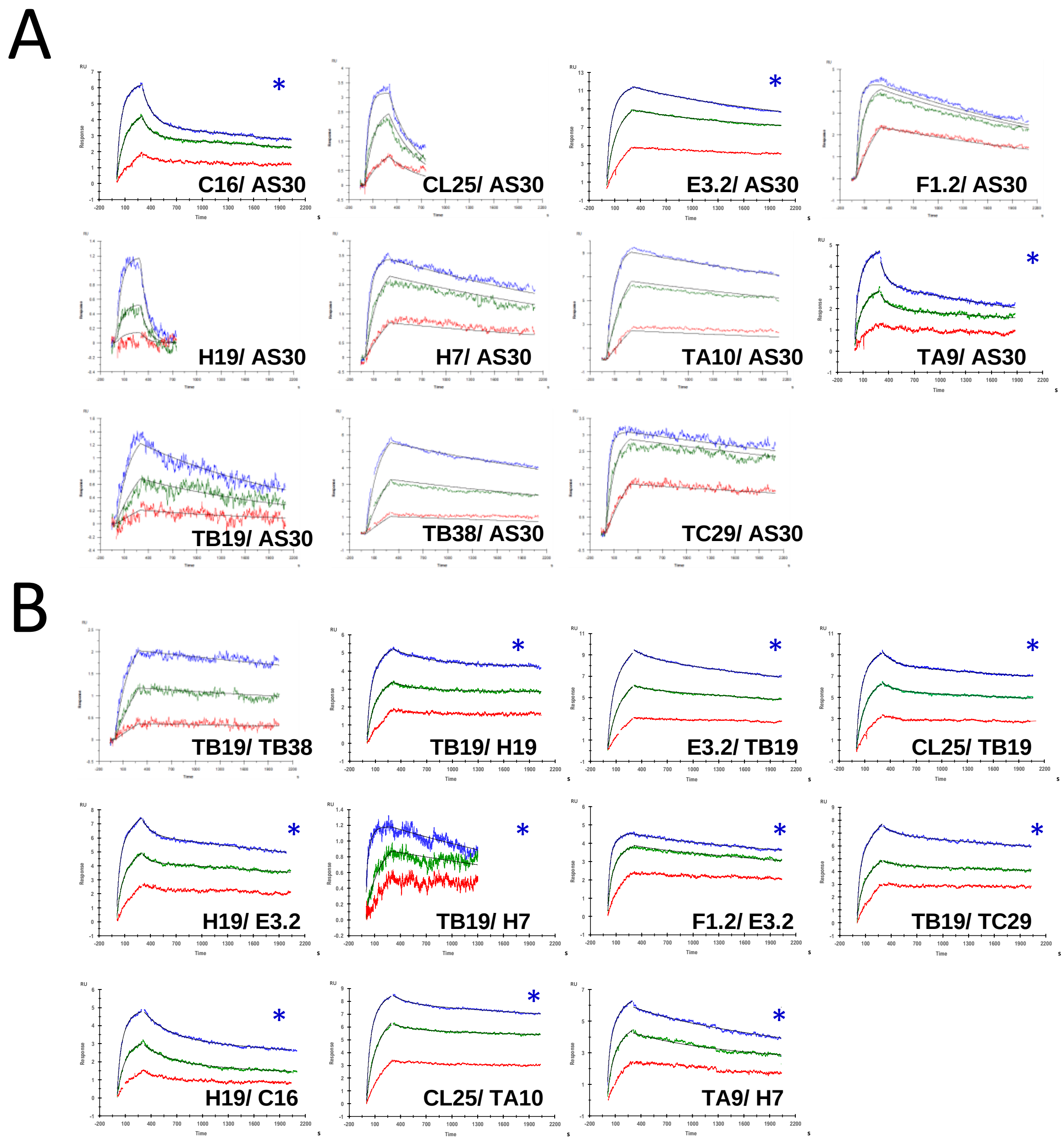


**Figure S8.** Fits to Biacore SPR data to assess bivalent binding of biparatopic antibodies to CD73. Some data could be described by single-component binding and dissociation and were fitted using a 1:1 Langmuir binding model with BiaEvaluation software. Several (indicated by asterisks) showed biphasic association and dissociation kinetics. In those cases fits were performed at two concentrations of CD73 (32nM and 12nM, blue and green respectively) using a reiterative process to derive abundances and rate constants for each component during association and dissociation as described in Methods. Best fit lines are shown in black. The rate constants obtained by both approaches are presented in Table S2. **A**. Monovalent parental antibodies. **B**. Biparatopic antibodies.

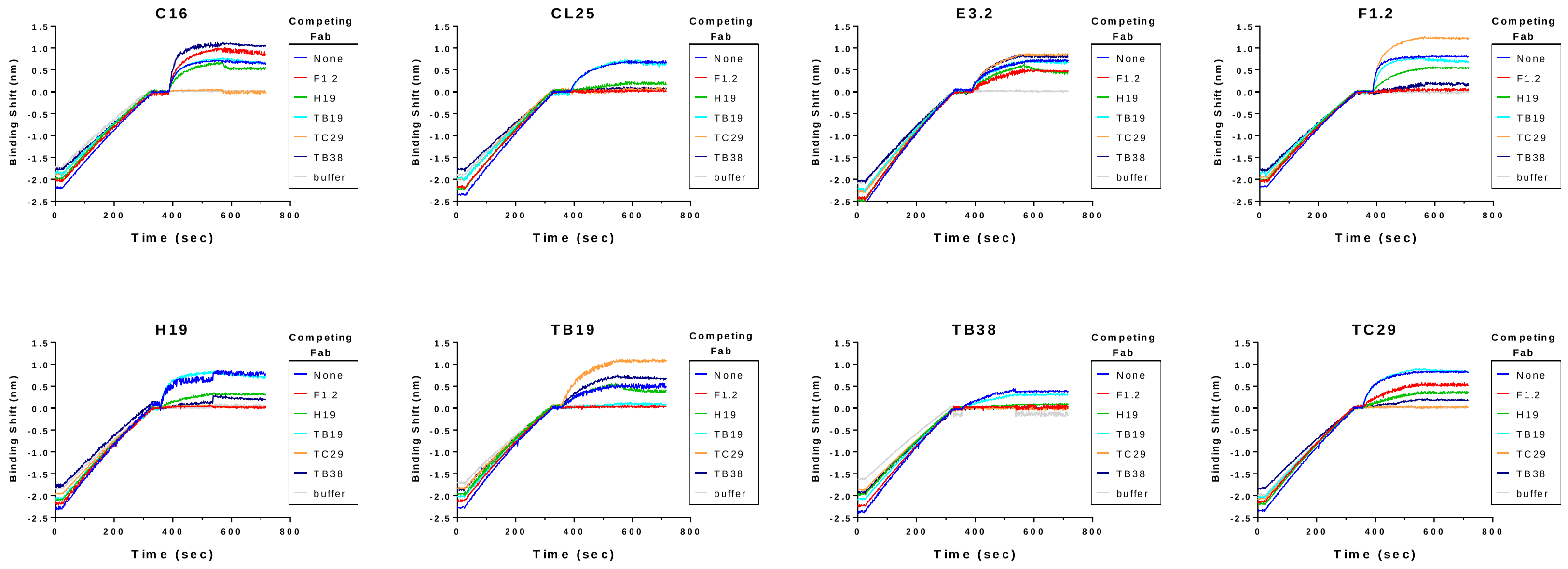


**Figure S9**. Epitope mapping by a premix competition approach. The capture of Fab:CD73 complexes in solution by monovalent CD73 antibodies on protein A biosensor tips was followed by Octet. Titles: name of antibody loaded on the tips. Sensorgram colors identify the Fab preincubated with CD73 (see legend). Blue trace: CD73 alone. The initial rise for 300s reflects loading of the IgGs on the tips. Following washing, CD73 premixed with excess Fab was applied. Higher responses reflect greater mass capture. Note that preincubation with a Fab with an overlapping epitope will block capture and produce no change in the sensorgram. Capture of a CD73 bound by a Fab to a non-overlapping epitope will produce a higher signal than CD73 alone on account of the larger size of the complex. The binding shift values normalized to CD73 (Materials and Methods) are shown in Figure 3B.

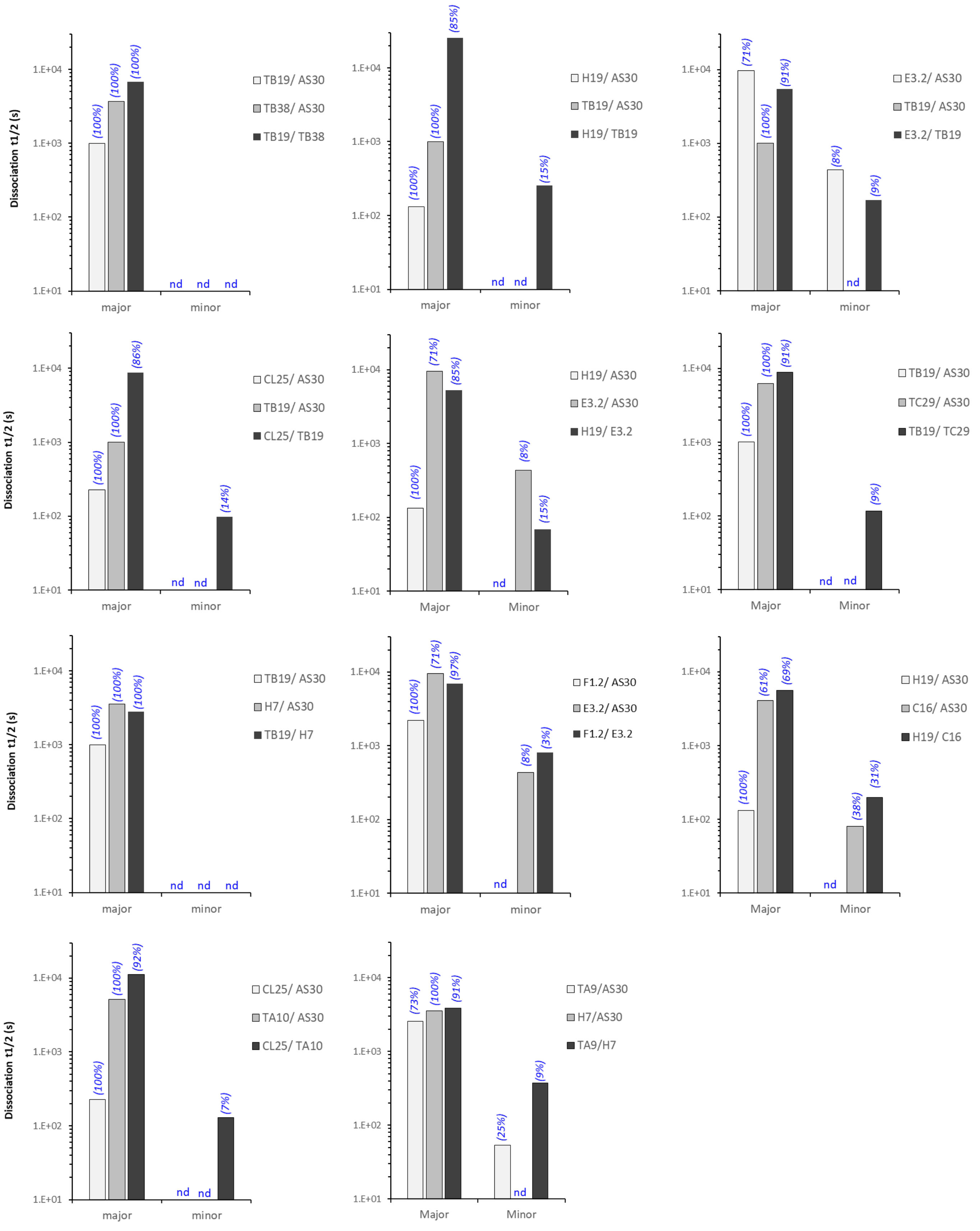


**Figure S10.** Dissociation half-times of CD73 from immobilized monovalent parent and biparatopic antibodies. Half times are calculated from rate constants presented in Table S2. Where biphasic kinetics were observed the major and minor components are shown separately and the fraction of each are shown in blue italics. nd: not detectable.
